# Interventions to address potentially inappropriate prescriptions and over-the-counter medication use among adults 65 years and older in primary care settings: protocol for a systematic review

**DOI:** 10.1186/s13643-022-02044-w

**Published:** 2022-10-20

**Authors:** Andrew Beck, Navindra Persaud, Laure A. Tessier, Roland Grad, Michael R. Kidd, Scott Klarenbach, Christina Korownyk, Ainsley Moore, Brett D. Thombs, Dee Mangin, Rita K. McCracken, Emily G. McDonald, Caroline Sirois, Salmaan Kanji, Frank Molnar, Stuart G. Nicholls, Kednapa Thavorn, Alexandria Bennett, Nicole Shaver, Becky Skidmore, Bradley R. Mitchelmore, Marc Avey, Elizabeth Rolland-Harris, Julian Little, David Moher

**Affiliations:** 1 School of Epidemiology and Public Health, Faculty of Medicine, University of Ottawa, Ottawa, Ontario Canada; 2 Department of Family and Community Medicine, St. Michael’s Hospital, Toronto, Ontario Canada; 3 Global Health and Guidelines Division, Public Health Agency of Canada, Ottawa, Ontario Canada; 4 Department of Family Medicine, McGill University, Montreal, Quebec Canada; 5 College of Health & Medicine, The Australian National University, Canberra, Australia; 6 Department of Family and Community Medicine, University of Toronto, Toronto, Ontario Canada; 7 Department of Medicine and Dentistry, University of Alberta, Edmonton, Alberta Canada; 8 Department of Family Medicine, University of Alberta, Edmonton, Alberta Canada; 9 Department of Family Medicine, McMaster University, Hamilton, Ontario Canada; 10 Lady Davis Institute of the Jewish General Hospital and Faculty of Medicine, McGill University, Montreal, Quebec Canada; 11 Department of General Practice, University of Otago, Dunedin, New Zealand; 12 Department of Family Practice, University of British Columbia, Vancouver, British Columbia Canada; 13 Department of Medicine, McGill University Health Centre, Montreal, Quebec Canada; 14 Faculty of Pharmacy, Laval University; Centre d’excellence sur le vieillissement de Québec, VITAM research Centre, Québec, Québec Canada; 15 The Ottawa Hospital, Ottawa Hospital Research Institute, Ottawa, Ontario Canada; 16 Faculty of Medicine, University of Ottawa, Ottawa, Ontario Canada; 17 Division of Geriatric Medicine, The Ottawa Hospital, The Ottawa Hospital Research Institute, Bruyere Research Institute, Ottawa, Ontario Canada; 18 Clinical Epidemiology Program, Ottawa Hospital Research Institute, Ottawa, Ontario Canada; 19 Independent Information Specialist, Ottawa, Ontario Canada

**Keywords:** Inappropriate prescribing, Over-the-counter medication, Polypharmacy, Systematic review, Older adults, Guideline, Primary health care

## Abstract

**Purpose:**

To inform recommendations by the Canadian Task Force on Preventive Health Care on potentially inappropriate prescribing and over-the-counter (OTC) medication use among adults aged 65 years and older in primary care settings. This protocol outlines the planned scope and methods for a systematic review of the benefits and harms and acceptability of interventions to reduce potentially inappropriate prescriptions and OTC medication use.

**Methods:**

De novo systematic reviews will be conducted to synthesize the available evidence on (a) the benefits and harms of interventions to reduce potentially inappropriate prescriptions and OTC medications compared to no intervention, usual care, or non- or minimally active intervention among adults aged 65 years and older and (b) the acceptability of these interventions or attributes among patients. Outcomes of interest for the benefits and harms review are all-cause mortality, hospitalization, non-serious adverse drug reactions, quality of life, emergency department visits, injurious falls, medical visits, and the number of medications (and number of pills). Outcomes for the acceptability review are the preference for and relative importance of different interventions or their attributes. For the benefits and harms review, we will search MEDLINE, Embase, and Cochrane Central Register of Controlled Trials for randomized controlled trials. For the acceptability review, we will search MEDLINE, Embase, PsycInfo, Cochrane Central Register of Controlled Trials, and the NHS Economic Evaluation Database for experimental and observational studies with a comparator. Websites of relevant organizations, other grey literature sources, and reference lists of included studies and reviews will be searched. Title and abstract screening will be completed by two independent reviewers using the liberal accelerated approach. Full-text review, data extraction, risk of bias assessments, and GRADE (Grading of Recommendations Assessment, Development and Evaluation) will be completed independently by two reviewers, with any disagreements resolved by consensus or by consulting with a third reviewer. The GRADE approach will be used to assess the certainty of the evidence for outcomes.

**Discussion:**

The results of this systematic review will be used by the Canadian Task Force on Preventive Health Care to inform their recommendation on potentially inappropriate prescribing and OTC medication use among adults aged 65 years and older.

**Systematic review registration:**

PROSPERO (KQ1: CRD42022302313; KQ2: CRD42022302324); Open Science Framework (https://osf.io/urj4b/).

**Supplementary Information:**

The online version contains supplementary material available at 10.1186/s13643-022-02044-w.

## Background

Prescription and over-the-counter (OTC) medication use can be defined as appropriate, where it has been optimised, prescribed or used according to the best evidence, with patient values and goals incorporated in the treatment plan, and follow-up confirms the intended benefit is achieved. Medication use defined as either inappropriate or problematic would reflect the opposite and can involve single or multiple medications. Inappropriate prescriptions may include overprescribing (where more medications are prescribed than clinically needed, or are prescribed for longer that clinically needed), misprescribing (incorrectly prescribing a medication, e.g., wrong dosage or frequency), or inappropriate combinations (incorrect combinations of multiple medications that cause cumulative burden) [1–4].

### Prevalence of prescription and over-the-counter medication use among older adults

A Canadian report using data from the National Population Health Survey found that older adults were major consumers of prescription medications, OTC medications, and natural products [5]. In 2016, a Canadian Institute for Health Information report reviewing all provincial and Yukon drug claims among older adults from the National Prescription Drug Utilization Information System (NPDUIS) found that 66% of adults in Canada aged 65 years and older were prescribed cumulatively five or more different drug classes over a year and 27% were prescribed ten or more [6]. Further, 35% of older adults had reported the use of five or more different drug classes in the long-term; long-term use was defined as at least two drug claims and a 180-day cumulative supply over 1 year [6]. Polypharmacy prevalence data in Canada may be underestimated given that OTC medications are rarely reported or studied. One article stated that the average OTC usage among older American adults was four OTC medications, however, no details on the data or study were referenced. It has been estimated that approximately 25% of older American adults were using a combination of 10 or more prescription and OTC medications [7, 8].

### Burden of inappropriate prescription and over-the-counter medication use among older adults

Potentially inappropriate prescriptions and OTC medication use among adults aged 65 years and older are commonly associated with negative health outcomes, adverse drug events (including postural hypotension, falls, injuries leading to hospitalization among others), increased risk of frailty, disability, morbidity, mortality, and reduced health-related quality of life and activities of daily living [6, 9–19]. The risk of adverse events is likely to increase with rising levels of frailty, multi-morbidity, and functional decline among older adults [20, 21]. Medication regimens for older people are often complex and can be challenging with the increased presence of various chronic conditions and multiple therapies (prescribed or non-prescribed) [22–24]. A national study using telephone survey data of 3132 older adults from the 2008 Canadian Survey of Experiences with Primary Health Care found that within the past year, 12% of Canadian older adults on five or more prescription medications had experienced an adverse medicine reaction that required a medical doctor or emergency room visit [25]. Also, 3% of older adults taking prescription medications reported receiving the wrong medication or dose from their healthcare provider, with 39% of those resulting in “somewhat serious” or “very serious” problems [25].

Furthermore, polypharmacy (commonly defined as the concomitant use of five or more medications, however, there is no consensus on the definition and various interpretations exist [26]) is frequent in populations with multi-morbidities and while many medications may be identified to offer some effectiveness in each single disease case, the combined prescribed or OTC medication use may cause harm exceeding benefit overall [7, 9, 27, 28]. Inappropriate prescribing can also occur with single medications that may provide patients with limited benefit while increasing the risk of side effects or potential harms (e.g., routine aspirin use among older adults with low cardiovascular disease risk and risk of gastrointestinal bleeding) [29–31]. The potential for inappropriate prescription medication use in older populations is further compounded by the addition of purchased medications without the need for a prescription, commonly referred to as OTC medications [7, 32]. These medications are used to treat common symptoms (e.g., heartburn, insomnia) and may introduce an additional layer of complexity to inappropriate drug use. OTC medication misuse is difficult to identify as patients can purchase and use OTC medications without their health care providers’ awareness [33].

### Assessing inappropriate prescription and over-the-counter medication use

Assessing if the use of prescription and OTC medication is inappropriate is complex because medications need to be assessed based on their indication, efficacy, and harms in relation to a patient’s medication profile and comorbidities and their individual experience of the benefits and harms, as well as their preferences and life circumstances (e.g., financial situation, ability to manage a complex regime, predicted lifespan), and the availability of alternative pharmacological and non-pharmacological medications treatment [34]. Such complexity in assessing inappropriate or problematic prescribing requires some operationalization.

### Interventions to detect potentially inappropriate prescribing and OTC medication use

There are two approaches, explicit and implicit. Explicit approaches involve lists of “drugs to avoid,” related to the most common adverse drug reactions in older adults and explicit interventions aim to identify these specific potentially inappropriate medications or medication combinations that are criterion-based.

One example of a common explicit approach is the *American Geriatrics Society Beers Criteria for Potentially Inappropriate Medication Use in Older Adults* [11, 35, 36], a list of potentially inappropriate medications that should generally be avoided in older adults. The Beers Criteria was developed in 1991, based initially on expert panel consensus, with systematic review evidence subsequently added. The latest version was published in 2019 [36]. It is widely used in North America by clinicians, educators, researchers, healthcare administrators, and regulators including in Canada (e.g., Health Canada). Following the Beers initiative in 1991, other country-specific lists were established: in the USA, Assessing Care of Vulnerable Elders (ACOVE), Geriatric Risk Assessment MedGuide (GRAM); in Ireland, the STOPP (Screening Tool of Older Persons' Prescriptions) and START (Screening Tool to Alert to Right Treatment) criteria; in Canada, the Improving Prescribing in the Elderly Tool (IPET); in Germany, PRISCUS (Latin for ‘old and venerable’) and the Fit for the Aged (FORTA); in France, a consensus panel list; and in Norway, the Norwegian General Practice (NORGEP) list have also been developed. Other scales specifically identify the anticholinergic burden of medications—with the Drug Burden Index, which includes the sedative burden in addition to the anticholinergic burden, appearing to best predict adverse health outcomes [37, 38].

Validated screening tools could assist clinicians in identifying inappropriate prescribing and OTC medication use in older adults [39]. However, these approaches do have some limitations. For example, and Beers Criteria acknowledges this, the lists are limited to drugs causing the most common adverse events and do not consider cost, availability, the complexities of patient preferences, and cumulative serious adverse event burdens as well as interactions. Therefore, the utility is limited unless used as part of an implicit approach.

Implicit interventions aim at identifying potentially inappropriate medications in general and rely on expert professional judgement while focusing on the patient’s individual circumstances and priorities and addresses the entire medication regimen (e.g., CRIteria to assess appropriate Medication use among Elderly complex patients [CRIME], Palliative and Therapeutic Harmonization [PATH], Medication Appropriateness Index [MAI]) [40, 41]. However, these implicit approaches are more complex, and while more suited to multi-morbidity, are less studied. Additionally, interventions for detecting inappropriateness can involve several sources of complexity [42]. For example, interventions may include multiple components (e.g., screening tools, medication review, financial incentives for practices to review patients' charts to assess appropriateness), target certain populations or medications (e.g., those on five or more medications, or specific drug classes), be delivered by different healthcare providers and in different settings. Interventions may also involve implementation complexities such as financial incentives, electronic medical record alerts, or supplemental training [43]. At least 15 different tools to identify problematic or inappropriate prescribing are available to clinicians [13], with numerous national, provincial, organizational tools available as guidance in Canada [44–46].

Therefore, evidence-based guidelines on potentially inappropriate prescribing could help improve patient outcomes by facilitating both implicit and explicit approaches, where first contact care providers and patients share decision-making on the appropriateness of medications [39].

### Rationale

This systematic review seeks to determine the benefits and harms of interventions delivered by, or initiated by, first contact care providers to reduce potentially inappropriate prescriptions and potentially inappropriate OTC medication use in adults aged 65 years and older. The results will be used by the Canadian Task Force on Preventive Health Care (task force) to inform their recommendations on reducing potentially inappropriate prescribing and OTC medication use among adults aged 65 years and older in relevant primary care settings.

## Methods

### Protocol development

This protocol was developed by the Evidence Review and Synthesis Centre (ERSC) at the University of Ottawa in consultation with the working group (which consists of some of the Task Force members), external clinical experts, staff from the Global Health and Guidelines Division at the Public Health Agency of Canada, other Task Force members not part of the working group, and stakeholder organizations. The working group formulated and finalized the key questions (KQs) and PICOS (population, interventions, comparators, outcomes, and setting/study design) for each KQ with involvement from the ERSC. The working group, external clinical experts, and Global Health and Guidelines Division staff will not be involved in the selection of studies, data extraction, or data analysis, but will be consulted for advice, when required, with all final decisions made by the ERSC.

Reporting of the protocol was guided by the Preferred Reporting Items for Systematic Reviews and Meta-Analyses Protocols (PRISMA-P) checklist [47] (Additional file 1). The project will be developed, conducted, and prepared according to the Task Force Methods Manual [48], with guidance from the Cochrane Handbook [49]. The systematic review will be reported using the Preferred Reporting Items for Systematic Reviews and Meta-Analyses on Complex Interventions (PRISMA-CI) checklist [50]. A preprint version of this protocol was publicly available through the journal on ResearchSquare. The protocol was registered in the PROSPERO database (KQ1: CRD42022302313; KQ2: CRD42022302324). Any amendments to the protocol will be documented in the PROSPERO registration and the final published report.

### Key questions and objective

Key question 1 (KQ1): What are the *benefits and harms* of interventions to reduce potentially inappropriate prescriptions and over-the-counter medication use in adults aged 65 and older?

Key question 2 (KQ2): What is the *acceptability* of interventions to address potentially inappropriate prescriptions and over-the-counter medication use in adults aged 65 and older?

**Fig. 1:**
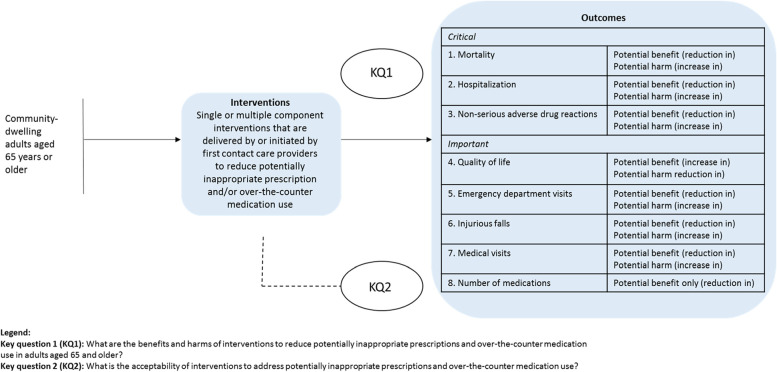
Analytic framework

A staged approach will be undertaken to address both KQs. During the first stage, we will synthesize the available clinical trial evidence on the effectiveness of interventions to address potentially inappropriate prescriptions and use of OTC medications among adults aged 65 years and older (KQ1). If evidence from KQ1 indicates that an intervention or attributes of an intervention are effective for at least one of the critical outcomes (i.e., all-cause mortality, hospitalization, non-serious adverse drug reactions), then stage 2 will examine the acceptability of these interventions or attributes among patients (KQ2). Acceptability indicators will include patient preferences and relative importance (e.g., actual participation, preferring one type of intervention or attribute, intentions to participate, the strength of associations between attribute ranking/ratings, and behaviors or intentions for interventions). Figure 1 presents the analytic framework of the KQs, relevant population, interventions, and outcomes to be considered.

### Eligibility criteria

Eligibility criteria were established by the working group using the PICOS framework with input from clinical experts external to the working group, and the ERSC. The inclusion and exclusion criteria for KQ1 and KQ2 are listed in Tables 1 and 2 and described below. Table 3 provides examples of potential interventions and Table 4 presents the final set of benefit and harm outcomes deemed to be of critical or important for guideline development and decision-making

**Table 1:** Key question 1 eligibility criteria

Criteria	Inclusion	Exclusion
**Population**	Community-dwelling adults aged 65 years or older. Studies recruiting adults under the age of 65 may be included if one of the following applies: - > 80% of the study sample are aged 65 years and older; - Participants’ mean age minus one standard deviation is equal to or greater than 65; - Results for adults 65 years and older are provided separately in stratified randomized studies.	Studies focussed only on participants < 65 years old.
**Intervention**	Single or multiple component interventions that are delivered by or initiated by first contact care providers (such as family physicians, general practitioners, pharmacists, nurses, geriatricians, physician assistants) to reduce potentially inappropriate prescription and/or over-the-counter medication use (e.g., medication review, electronic decision support system, patient/clinician education). Interventions may be targeting one specific drug or drug class, or multiple drugs and drug classes (drug[s] must be assigned a Drug Identification Number (DIN) or international non-proprietary name (INN)).	Interventions focussed only on complementary alternative medications.
**Comparator**	No intervention, usual care, non- or minimally-active intervention.	Single or multiple component interventions to reduce potentially inappropriate prescription and/or over-the-counter medication use.
**Outcomes**	Critical/primary 1. All-cause mortality (benefit or harm) 2. Hospitalization (benefit or harm) 3. Non-serious (i.e., not requiring hospitalization and not causing death) adverse drug reactions including those related to withdrawal or stopping medication (benefit or harm) Important/secondary 4. Quality of life (benefit or harm) 5. Emergency department visits (benefit or harm) 6. Injurious falls (benefit or harm) 7. Medical visits (benefit or harm) 8. Number of medications (and number of pills) (benefit) *Outcomes all-cause mortality, hospitalization, non-serious adverse drug reactions, quality of life, emergency department visits, injurious falls and medical visits may be considered as a benefit or harm of the intervention depending on the direction of effect.*	
**Setting**	Primary care; Settings relevant to primary care where intervention is delivered by or initiated by first contact care providers (such as home, community, nursing/long-term care homes, pharmacy, emergency department).	Studies focussed on settings not relevant to primary care (e.g., acute care inpatient setting, specialist clinics).
**Study design**	Randomized controlled trials (RCTs) (individual or cluster)	Systematic reviews, non-randomized controlled trials, observational study designs (e.g., cohort studies), descriptive study designs (e.g., case reports, case series). Letters, commentaries, editorials.
**Publication language**	English or French	Languages other than English and French
**Dates of publication**	No year limitation

**Table 2:** Key question 2 eligibility criteria

Criteria	Inclusion	Exclusion
**Population**	Community-dwelling adults aged 65 years or older. Studies recruiting adults under the age of 65 may be included if one of the following applies: - > 80% of the study sample are aged 65 years and older; - Participants’ mean age minus one standard deviation is equal to or greater than 65; - Results for adults 65 years and older are provided separately.	Studies focussed only on participants < 65 years old.
**Exposure/intervention**	Experience with interventions to reduce use of potentially inappropriate prescribed and/or over-the-counter medications, or exposure to information about different types and/or attributes of interventions (e.g., mode, duration, setting, delivery providers, type of intervention). Study must relate to types of interventions or attributes of interventions shown to be effective for at least one critical/primary outcome, from KQ1.	
**Comparator**	Depending on the study design, comparator may be: a) Experience with different types of intervention, or b) Information about a different type of intervention, in terms of its components and/or attributes	
**Outcomes**	Depending on study design: Preference for different interventions or their attributes Quantitative information about relative importance of different interventions or their attributes (e.g., actual participation, proportion preferring one type of intervention or attribute, intentions to participate, dropouts, strength of associations between attribute ranking/ratings and behaviors)	
**Setting**	Any relevant setting to primary care where intervention is delivered by or initiated by first contact care providers (e.g., primary care, home, community, nursing/long-term care homes, emergency department)	Settings not relevant to primary care or not targeting general community-dwelling population (e.g., workplaces, inpatient settings, specialist settings)
**Study design**	RCTs (individual or cluster), non-randomized experimental studies (i.e., study assigned intervention without randomized allocation), or observational study design with a comparator reporting stated preferences or relative importance using the methods described below: a) Preference measured directly via conjoint analysis with choice experiments or probability trade-offs (e.g., best-worst scaling choice experiment) b) Surveys/questionnaires or studies evaluating decision aids	Systematic reviews, case-control studies, case reports, case series, qualitative studies. Letters, commentaries, editorials.
**Publication language**	English or French	Languages other than English and French
**Dates of publication**	No year limitation

**Table 3:** Examples of interventions for addressing potentially inappropriate prescribing and/or OTC medication use

Intervention	Description
Audit and feedback	The process of providing a summary of clinical performance of healthcare provider over a specified period. Provides data showing discrepancies between current and target performance and can include comparison of individual performance in relation to other health professionals.
Computerized software tool	A computer program that scans the patient’s electronic medical record to identify current (and/or previous) over-the-counter and prescription drugs. This includes simple interaction checkers in most prescription software as well as more focused tools.
Computerised decision support	Electronic tools that prompt healthcare provider behaviors in various areas of patient care, including medication ordering and chronic disease management.
Education programs	Programs that educate healthcare providers and prescribers about the benefits and risks of prescribing or education sessions with the intention to reduce medication use.
Medication reconciliation	The process of comparing what a patient has been prescribed by a healthcare provider to what they are actually taking. This is done to avoid medication errors and may aid in deprescribing some drugs.
Medication review	The process by which a healthcare provider examines the patient or participant’s current (and/or previous) list of OTC and prescription drugs. This can be facilitated with a computerized program. This may be an explicit review, focused on “drugs to avoid” or an implicit review (integrating broader aspects of the patient context and experience).

**Table 4:** Final set of benefit and harm outcomes deemed to be of *critical* or *important* for guideline development and decision-making

Outcome	Consideration of the benefits/harms associated with the preventive health care intervention	Priority
All-cause mortality	• Potential benefit (reduction in) • Potential harm (increase in)	Critical
Hospitalization	• Potential benefit (reduction in) • Potential harm (increase in)	Critical
Non-serious (i.e., not requiring hospitalization and not causing death) adverse drug reactions including those related to withdrawal or stopping medication	• Potential benefit (reduction in) • Potential harm (increase in)	Critical
Quality of life	• Potential benefit (increase in) • Potential harm (reduction in)	Important
Emergency department visits	• Potential benefit (reduction in) • Potential harm (increase in)	Important
Injurious falls	• Potential benefit (reduction in) • Potential harm (increase in)	Important
Medical visits	• Potential benefit (reduction in) • Potential harm (increase in)	Important
Number of medications (and number of pills)	• Potential benefit only (reduction in)	Important

### Key question 1

#### Population

Our population will include adults aged 65 years and older residing in the community, or nursing or long-term care homes. While this systematic review will inform a guideline targeting adults aged 65 and older residing in the community, studies conducted in nursing or long-term care homes will be included if their studied interventions are applicable to community-dwellers in a primary care setting. This age range was selected because co-morbidities and overprescribing issues are more common in this population and because adverse effects are more likely [6, 25, 51].

#### Intervention

Any intervention, alone or in combination, for reducing the use of potentially inappropriate prescription drugs (i.e., drugs that require a prescription from a healthcare provider) and/or potentially inappropriate OTC medication (i.e., drugs that may be obtained directly in a pharmacy without a prescription) will be considered for study inclusion. The intervention should be delivered by or initiated by first contact care providers and may target one specific drug or drug class, or multiple drugs and drug classes; drug(s) must be assigned a Drug Identification Number (DIN) or international non-proprietary name (INN). We will include studies from relevant settings to primary care including primary care, home, community, nursing, or long-term care settings. Recommendations will be made for primary care providers, considering the effectiveness of interventions in, or initiated by, any first contact care setting. Examples of potential interventions are outlined in Table 3.

#### Comparators

Eligible comparator groups within studies will include no intervention, usual care, or a non- or minimally active intervention.

#### Outcomes

Members of the working group developed a list of preliminary outcomes of interest. Through consensus, those outcomes were rated by the five working group members according to GRADE methodology as critical (rated 7 to 9 out of 9), important (rated 4 to 6 out of 9), or of limited importance (rated 1 to 3 out of 9) for making guideline recommendations [52]; only critical and important outcomes will be included in the systematic review. For more details on the outcome rating process, please see https://canadiantaskforce.ca/methods/.

Based on ratings and discussion by the five working group members, outcomes of interest deemed of critical importance for guideline development and decision-making are all-cause mortality (mean rating 7.8), hospitalization (mean rating 7.6), and non-serious (i.e., not requiring hospitalization and not causing death) adverse drug reactions including those related to withdrawal or stopping medication (mean rating 7.0). Outcomes rated as important are quality of life (mean rating 6.8), emergency department visits (mean rating 6.4), injurious falls (mean rating 6.2), medical visits (mean rating 5.4), and the number of medications (and number of pills) (mean rating 4.0). The outcomes may be considered as a benefit or harm of the intervention as reported by authors (see Table 4).

#### Study design

Only randomized controlled trials (RCTs) will be included.

### Key question 2

#### Population

Our population will include adults aged 65 years and older residing in the community, nursing, or long-term care homes. While this systematic review will inform a guideline targeting adults aged 65 and older residing in the community, acceptability of older adults residing in nursing or long-term care homes will also be considered in this systematic review. Acceptability in these populations could be generalized to adults aged 65 years and older residing in the community.

#### Exposure/intervention

We are interested in the patients’ experience with the intervention or attributes of the intervention deemed effective from KQ1 or exposure to information about the different types and/or attributes of the interventions (e.g., mode, duration, setting, delivery providers, type of intervention).

#### Comparators

Depending on the study design, eligible comparator groups within studies will include no comparison, experience with different types of intervention, or information about a different type of intervention in terms of its components and/or attributes.

#### Outcomes

Primary outcomes will be the preference for and relative importance of different interventions or their attributes (e.g., actual participation, proportion preferring one type of intervention or attribute, intentions to participate, dropouts, the strength of associations between attribute ranking/ratings and behaviors, and quantitative information about relative importance of different interventions or their attributes.

#### Study design

The following study designs will be eligible for inclusion: experimental and observational studies with a comparator (i.e., RCTs, quasi-randomized controlled trials, non-randomized controlled trials) that report the populations’ stated preferences or relative importance concerning the effective interventions or attributes. Case-control studies, case series, case reports, and qualitative studies will be excluded.

### Information sources and search strategy

For both KQs, the search strategies have been developed and tested through an iterative process by an experienced medical information specialist (BS) in consultation with the review team. The MEDLINE strategies for each question were peer-reviewed by another senior information specialist using the PRESS Checklist [53].

### Key question 1

We will conduct the searches using the OVID platform, and will search Ovid MEDLINE® ALL, Embase Classic+Embase, and Cochrane Central Register of Controlled Trials. Strategies utilized a combination of controlled vocabulary (e.g., “polypharmacy,” “inappropriate prescribing,” “health services for the aged”) and keywords (e.g., “polyprescribing,” “deprescribing,” “geriatric”). An RCT filter has been applied to the MEDLINE and Embase search strategies and is restricted to adults aged 65 and older. There are no language or date restrictions in the search strategies. Animal-only records and opinion pieces will be removed from the conducted search results. The search strategies are available in Additional file 2.

### Key question 2

We will conduct the searches using the OVID platform, and will search Ovid MEDLINE® ALL, Embase Classic+Embase, APA PsycInfo, Cochrane Central Register of Controlled Trials, and the NHS Economic Evaluation Database. Strategies utilized a combination of controlled vocabulary (e.g., “polypharmacy,” “health services for the aged,” “patient participation”) and keywords (e.g., “polyprescribing,” “geriatric,” “patient engagement”). There are no language or date restrictions in the search strategies. Animal-only records will be removed from the conducted search results. The search strategies are available in Additional file 2.

Literature saturation will be ensured by supplementing the electronic database searches with grey literature sources and reviewing the bibliographies of included trials and other relevant evidence-based clinical practice guidelines and systematic reviews. The following criteria will be used to consider systematic reviews [54]: (1) at least one database was searched; (2) study selection criteria were reported; (3) risk of bias of included studies was reported; and 4) a list and synthesis of included studies was reported. We will contact authors (by email with a maximum of three attempts) of relevant conference abstracts and protocols for manuscripts or unpublished data. We will search grey literature sources for unpublished documents using the Canadian Agency for Drugs and Technologies in Health (CADTH) Grey Matters checklist [55]. Searches will be limited to English and French language documents. In addition to the CADTH checklist, we will search websites of relevant organizations as suggested by the working group and clinical experts. The full list of relevant websites is available in Additional file 3.

### Study selection

Before the screening process, records retrieved from the literature searches will be uploaded to Covidence, an online systematic review management software package [56]. To ensure high inter-rater reliability, we will conduct pilot exercises before title and abstract screening and full-text review using the pre-determined eligibility criteria for each KQ. Standardized screening forms for study selection will be developed and tested on a random sample for 50 titles and abstracts and 25 full-text articles by reviewers. Any discrepancies among reviewers will be resolved by discussion or consulting with a third reviewer. Adjustments to the form will be made as needed.

Title and abstract screening will be completed in random order by two reviewers using the liberal accelerated approach [57]. All citations labelled by one reviewer as “included” or “unsure” will move forward to full-text review. The second reviewer will verify the excluded citations considered by the first reviewer to confirm their exclusion. The references for the second reviewer will be randomized to ensure they do not know whether the citation is a first review or verification. Conflict resolution will not be required at this screening stage. Full-text review will be completed in duplicate by pairs of reviewers screening independently. Any discrepancies will be resolved by consensus among the two reviewers or by a third reviewer.

We will order articles that are not available electronically through the interlibrary loan service. Where only abstract information is available, articles will be included if sufficient information is provided. If a potentially relevant study reports information that is unclear for a decision on eligibility, the corresponding author will be contacted for additional information twice by email over 1 month. If no response is received, the article will be excluded. For the excluded articles, the reasons for exclusion will be agreed upon by reviewers and a list of excluded studies with reasons will be provided in the final manuscript. If advice is required on potentially eligible studies, we will consult with the working group and clinical experts on the study design and outcomes collected. Attempts will be made to anonymize the article to avoid study identification and outcome data. The ERSC will decide on the eligibility of these studies.

### Data extraction

Prior to data extraction, we will develop a standardized extraction form and conduct a pilot exercise with two reviewers in Covidence on a random sample of five articles [56]. Any discrepancies between reviewers will be resolved by discussion or consulting with a third reviewer and adjustments to the form will be made as needed. The data extraction stage will be completed independently by two reviewers, with any disagreements in extractions resolved by consensus or by consulting with a third reviewer if consensus cannot be reached.

We will extract information on study characteristics (e.g., author, year of publication, country, study design, eligibility criteria), healthcare provider characteristics (e.g., type, discipline, specialty training), participant characteristics (e.g., age, number of medications/pills, comorbidities), setting (e.g., primary care practice, pharmacy, long-term care), interventions/comparator examined (e.g., type and details of the intervention[s]/comparator, attributes of the intervention[s], drug classes targeted [if applicable]), explicit and/or implicit intervention, screening tool if used (e.g., Beers Criteria, STOPP/START criteria), outcomes of interest, adjudication method, timeframe, and results. The outcomes all-cause mortality, hospitalization, non-serious adverse drug reactions, quality of life, emergency department visits, and injurious falls may be considered benefits versus harms or vice versa, and we will utilize the authors’ reporting of these outcomes as a benefit or harm. Data as reported in the included studies will be re-formatted and presented in the text and tables of the final manuscript, as appropriate.

Since the interventions can be simple or complex, we will classify the interventions by considering both delivery level (patient vs. prescriber-, or population-level intervention) and coverage (explicit and/or implicit intervention). A patient-level intervention is where the providers or prescribers identified target medications or specific populations to address potentially inappropriate medication use and implemented the process with a patient or participant. A prescriber-level or population-level intervention is one that is delivered to a population of providers or prescribers, and the same intervention is delivered to every member of this population. For example, education interventions to prescribers [58].

An explicit intervention aims to identify specific medications or medication combinations that might be inappropriate, and that is criterion-based (e.g., list of drugs, drugs classes and dosages or cumulative side effect burdens known to cause harmful effects, Beers Criteria, Improved Prescribing in the Elderly Tool [IPET] (or McLeod), STOPP/START criteria, PRISCUS, Drug Burden Index, Anticholinergic Burden indices). An implicit intervention aims at identifying potentially inappropriate medications in general, and that relies on expert professional judgement while focusing on the individual patient context and priorities and addresses the entire medication regimen. For example, the MAI, Lipton Criteria or statements like “is there an indication for the drug?” [41]. Implicit interventions may use explicit tools.

Where information is missing or unclear, we will contact the authors of the study for additional information twice by email over 1 month. If there are multiple publications of the same study, we will extract data from the most recent publication and older publications will be used as secondary sources.

### Risk of bias assessment

Before study appraisal, two reviewers will pilot the criteria of each tool on a random sample of five included studies and the appropriate study design for the tool. Any conflicts will be resolved by discussion or the involvement of a third reviewer. Two reviewers will independently appraise study-level or outcome-level, where appropriate, risk of bias using the appropriate tool for the included studies. Any disagreements in the assessments will be resolved by consensus or by consulting with a third team member. The risk of bias assessments for each study will be used to inform the study limitations domain of the certainty of evidence assessment [59].

For KQ1, we will use the original Cochrane Risk of Bias Tool for Randomized Controlled Trials (version 1.0) as suggested by the 2014 Task Force manual [60]. Certain domains of the tool are outcome-specific (e.g., blinding of outcome assessors) and these will be assessed at the outcome level [49]. For outcome and analysis reporting bias, we will use the methods outlined in the Agency for Healthcare Research and Quality guidance [61]. For cluster randomized trials, we will assess recruitment bias in the “other sources of bias” domain of the Cochrane tool [62]. Recruitment bias can occur when participants are recruited after the randomization of clusters (or group of individuals), which could affect the types of participants recruited due to the awareness of intervention and control clusters [62]. We will categorize the overall risk of bias as low if all domains were assessed as low risk, high if at least one domain was assessed at high risk, or unclear if at least one domain was assessed as unclear risk of bias and no domain was at high risk.

For KQ2, we will use the Cochrane Risk of Bias Tool for Randomized Trials [60] for controlled trials, the Newcastle-Ottawa Scale for assessing cohort studies [63], the EPOC tools for controlled before-after studies and interrupted time series studies [64], and the NIH National Heart, Lung, and Blood Institute tool for cross-sectional studies [65].

### Synthesis of included studies

We will describe and present in tables the study characteristics, participant characteristics, intervention and comparator details, outcome results, and quality appraisals for the included studies. Where required, we will transform data from the included studies to ensure consistent presentation and synthesis of the results across studies. We will consider clinical (e.g., patient characteristics) and methodological (e.g., study design) heterogeneity of included studies prior to performing a meta-analysis. If study data are not appropriate for statistical pooling, we will describe the findings narratively and present the range of effects.

For cluster randomized trials, we will attempt to avoid unit-of-analysis errors when reporting the results and/or incorporating into meta-analysis. If available, we will use the intracluster correlation coefficient reported in the included trial in order to apply a design effect to the sample size and number of events in the treatment and control groups. If this is not reported, we will use an external estimate from similar studies.

We will assess statistical heterogeneity using the *I*^2^ statistic and Cochran’s *Q* test (threshold p-value < 0.10) and consider levels of low (0–25%), moderate (25–50%), substantial (50–75%), and considerable (> 75%) heterogeneity [66–70]. If appropriate, we will pool studies using the random-effects model using Review Manager version 5.4.1 (The Nordic Cochrane Centre, The Cochrane Collaboration, Copenhagen, Denmark). For KQ2, we will pool data from randomized controlled trials and observational studies separately. For dichotomous outcomes, we will report risk ratios or risk differences groups with corresponding 95% confidence intervals. For continuous outcomes, we will report mean differences with 95% confidence intervals. In cases where various measurement tools are used, we will report the standardized mean difference with 95% confidence intervals. We will follow GRADE guidance for calculating relative and absolute effects with 95% confidence intervals for the evidence profile tables and summary of findings. If considerable heterogeneity (defined as *I*^2^ statistic above 75%) is detected, we may decide not to combine data in a meta-analysis and will try to explain reasons for the heterogeneity via sensitivity analysis, and meta-regression.

For results from studies with low event rates (less than 1%), we will use the Peto odds ratio method. When group imbalances exist (e.g., control groups of unequal sizes), a large magnitude of the effect is observed, or when events are more frequent (5 to 10%), the Mantel-Haenszel method will be used [71].

When appropriate, we may conduct sensitivity analyses to assess the robustness of the results or to assess equity considerations. We may perform separate analyses restricting studies to those with low overall risk of bias, by publication type (e.g., removing abstracts only or preprints), or based on study design issues as considered in the risk of bias tool. We may also perform separate sensitivity analyses according to equity considerations (e.g., age, gender/sex, socioeconomic status), differing definitions of older adults by study authors, drug type targeted, intervention type, the number of medications/pills taken by the participants, how polypharmacy was defined by study authors, type of provider/setting (e.g., primary care practice, pharmacy, long-term care). However, other issues that we may want to examine through subgroup/sensitivity analyses may only be identified during the systematic review. All of these analyses are deemed exploratory in nature and should not be construed as a priori with definitive hypothesis.

We will follow previous guidance on meta-regression analyses [66]. The meta-regression analyses will be conducted when at least 10 studies are available for the outcome and intervention comparisons and will be based on random-effects models. We will use funnel plots and statistical tests (e.g., Egger regression test, Hedges-Olkin method) to assess for small-study effects (e.g., publication bias) [68, 72].

### Grading the certainty of evidence and interpretation

Two reviewers will independently appraise the certainty of the evidence using the GRADE approach and resolve disagreements by discussion or consulting a senior team member. Before the assessments, two reviewers will pilot GRADE assessments on a sample of two to three outcomes. The GRADE evidence profiles and summary of findings tables will be conducted for each outcome using the GRADE framework to assess each of the five domains (i.e., risk of bias, inconsistency, indirectness, imprecision, and publication bias) [48, 52]. For KQ2, we will perform GRADE assessments separately for different study designs. When conducting the assessments of the GRADE domains, the working group will be consulted to review the draft GRADE tables and determine the imprecision thresholds for interpreting the importance of effect sizes for each outcome. Afterwards, the GRADE assessments will be finalized by the ERSC, using the working group-selected thresholds. GRADE narrative statements will be prepared to represent the quantity, magnitude, and certainty of the evidence [73–75]. We will use the GRADEpro GDT online software to produce GRADE assessments and tables [76].

## Discussion

Findings from this project will inform the Task Force on the development of their evidence-based recommendations for primary care providers on interventions that reduce potentially inappropriate prescribing and OTC medication use in adults aged 65 years and older. The results of this project will be published in a peer-reviewed journal under the ‘Canadian Task Force on Preventive Health Care Evidence Reviews’ thematic series.

## Supplementary Information

**Additional file 1.** PRISMA-P 2015 checklist.

**Additional file 2.** Search strategies.

**Additional file 3.** List of grey literature relevant websites.

**Additional file 4.** Definitions.

**Additional file 5.** Stakeholder review and feedback.

## Data Availability

Project materials are available on the Open Science Framework (https://osf.io/urj4b/).

## Abbreviations

ERSC: Evidence Review and Synthesis Centre
GRADE: Grading of Recommendations Assessment, Development and Evaluation
KQ: Key questions
OTC: Over-the-counter
