# Supplementary material for: Interventions to address potentially inappropriate prescriptions and over-the-counter medication use among adults 65 years and older in primary care settings: protocol for a systematic review
Source: Syst Rev. 2022 Oct 20;11:225. doi: 10.1186/s13643-022-02044-w (PMC9585747; doi:10.1186/s13643-022-02044-w)
Supplement: Supplementary file 2 — Additional file 2. Search strategies. [file 13643_2022_2044_MOESM2_ESM.docx]

## Additional file 2: Search strategies

### Key question 1

Database: Embase Classic+Embase <1947 to 2020 July 22>, Ovid MEDLINE(R) ALL <1946 to July 22, 2020>, EBM Reviews - Cochrane Central Register of Controlled Trials <June 2020>

Search Strategy:

--------------------------------------------------------------------------------

1 exp polypharmacy/ (22068)

2 (polypharm* or poly-pharm*).tw,kf. (23876)

3 (polymedic* or poly-medic*).tw,kf. (1620)

4 (polyprescri* or poly-prescri*).tw,kf. (23)

5 (polypragmas* or poly-pragmas*).tw,kf. (279)

6 (deprescri* or de-prescri*).tw,kf. (2342)

7 ((cancel* or ceas* or cessation? or discontinu* or halt* or stop* or terminat*) adj2 (medicat* or medicine? or prescrib* or prescription?)).tw,kf. (20074)

8 ((cancel* or ceas* or cessation? or discontinu* or halt* or stop* or terminat*) adj (drug or drugs)).tw,kf. (2663)

9 Inappropriate Prescribing/ (7721)

10 ((appropriate* or inappropriate* or incorrect* or indiscriminat* or unnecessar* or rational* or irrational* or optimal* or optimum or suboptim* or sub-optim*) adj2 (medicat* or medicine? or prescrib* or prescription? or OTC or "over-the-counter" or "behind-the-counter")).tw,kf. (34820)

11 ((appropriate* or inappropriate* or incorrect* or indiscriminat* or unnecessar* or rational* or irrational* or optimal* or optimum or suboptim* or sub-optim*) adj (drug or drugs)).tw,kf. (20291)

12 ((excess* or multipl* or "five or more" or "5 or more") adj2 (medicat* or medicine? or prescrib* or coprescrib* or co-prescrib* or prescription? or coprescription? or co-prescription? or OTC or "over-the-counter")).tw,kf. (12059)

13 ((excess* or multipl* or "five or more" or "5 or more") adj (drug or drugs)).tw,kf. (20915)

14 ((concomitant* or concurrent*) adj2 (medicat* or medicine? or prescrib* or prescription? or OTC or "over-the-counter" or "behind-the-counter")).tw,kf. (18826)

15 ((concomitant* or concurrent*) adj (drug or drugs)).tw,kf. (4166)

16 ((omit* or omission?) adj2 (medicat* or medicine? or prescrib* or prescription? or drug or drugs)).tw,kf. (1715)

17 ((under$2 or over$2) adj2 (medicat* or prescrib* or prescription?)).tw,kf. (18025)

18 (underprescri* or overprescri*).tw,kf. (2820)

19 (quality adj2 (prescrib* or prescription?)).tw,kf. (2556)

20 ((multidrug? or multi-drug?) adj2 (pharmacotherap* or pharmaco-therap* or prescrib* or prescription? or regim* or therap* or treatment?)).tw,kf. (12410)

21 (multiple adj (pharmacotherap* or pharmaco-therap*)).tw,kf. (77)

22 ((medication? or medicine? or prescrib* or prescription?) adj3 cascad*).tw,kf. (229)

23 ((medication? or medicine? or prescrib* or prescription?) adj3 continuum*).tw,kf. (142)

24 ((medication? or medicine? or prescrib* or prescription?) adj3 legac*).tw,kf. (113)

25 ((medication? or medicine? or prescrib* or prescription?) adj3 (multi* chronic* or multi* comorbid* or multi* co-morbid* or multimorbid* or multi-morbid*)).tw,kf. (519)

26 (prescrib* adj3 (harm or harmed or harms or harming or harmful*)).tw,kf. (317)

27 STOPP.tw,kf. (1522)

28 "STOPP/START".tw,kf. (661)

29 ((Beers or McLeod or NPS) adj2 criteri*).tw,kf. (2084)

30 "Fit fOR The Aged".tw,kf. (246)

31 ((FORTA or RASP or Pricus) adj2 (criteri* or instrument? or list*)).tw,kf. (76)

32 Assessing Care of Vulnerable Elderly.tw,kf. (3)

33 ACOVE.tw,kf. (192)

34 Medication Appropriateness Index.tw,kf. (394)

35 Medication Regimen Complexity.tw,kf. (417)

36 Prescribing Optimi#ation Method.tw,kf. (6)

37 Systematic Tool to Reduce Inappropriate Prescribing.tw,kf. (29)

38 (strip adj5 (medication? or medicine? or prescrib* or prescription?)).tw,kf. (94)

39 or/1-38 [POLYPHARMACY] (193248)

40 Adult/ (12827729)

41 exp Aged/ (6489111)

42 aged.ti,kf. (152234)

43 ((age? or year?) adj2 ("65" or "66" or "67" or "68" or "69" or "70" or "71" or "72" or "73" or "74" or "75" or "76" or "77" or "78" or "79" or "80" or "81" or "82" or "83" or "84" or "85" or "86" or "87" or "88" or "89" or "90" or "91" or "92" or "93" or "94" or "95" or "96" or "97" or "98" or "99" or "100")).tw,kf. (1981078)

44 (elderly or geriatric* or gerontolog* or old-age? or senior?).tw,kf. (909167)

45 (older adj2 (adult* or female? or male? or man or men or patient? or person? or people? or population? or wom#n)).tw,kf. (533164)

46 (retiree? or retired or retirement).tw,kf. (46502)

47 (boomer? or babyboomer*).tw,kf. (3430)

48 Health Services for the Aged/ (54126)

49 Homes for the Aged/ (26688)

50 (old age adj (facilit* or home? or residen*)).tw,kf. (914)

51 or/40-50 [SENIOR FILTER] (16392895)

52 39 and 51 [POLYPHARMACY - SENIOR FILTER] (88361)

53 exp Animals/ not Humans/ (17515132)

54 52 not 53 [ANIMAL-ONLY REMOVED] (66924)

55 (comment or editorial or news or newspaper article).pt. (2094060)

56 (letter not (letter and randomized controlled trial)).pt. (2213083)

57 54 not (55 or 56) [OPINION PIECES REMOVED] (65812)

58 (controlled clinical trial or randomized controlled trial or pragmatic clinical trial or equivalence trial).pt. (1185459)

59 clinical trials as topic/ (303573)

60 exp Randomized Controlled Trials as Topic/ (328292)

61 (randomi#ed or randomi#ation? or randomly or RCT or placebo*).tw,kf. (3421922)

62 ((singl* or doubl* or trebl* or tripl*) adj (mask* or blind* or dumm*)).tw,kf. (693208)

63 trial.ti. (853918)

64 or/58-63 [RCT FILTER] (4330685)

65 57 and 64 [POLYPHARMACY - SENIORS - RCTs] (13512)

66 65 use medall [MEDLINE RECORDS] (4473)

67 polypharmacy/ (21763)

68 (polypharm* or poly-pharm*).tw,kw. (24987)

69 (polymedic* or poly-medic*).tw,kw. (1637)

70 (polyprescri* or poly-prescri*).tw,kw. (23)

71 (polypragmas* or poly-pragmas*).tw,kw. (284)

72 (deprescri* or de-prescri*).tw,kw. (2434)

73 ((cancel* or ceas* or cessation? or discontinu* or halt* or stop* or terminat*) adj2 (medicat* or medicine? or prescrib* or prescription?)).tw,kw. (20128)

74 ((cancel* or ceas* or cessation? or discontinu* or halt* or stop* or terminat*) adj (drug or drugs)).tw,kw. (2673)

75 exp inappropriate prescribing/ (9005)

76 ((appropriate* or inappropriate* or incorrect* or indiscriminat* or unnecessar* or rational* or irrational* or optimal* or optimum or suboptim* or sub-optim*) adj2 (medicat* or medicine? or prescrib* or prescription?)).tw,kw. (34986)

77 ((appropriate* or inappropriate* or incorrect* or indiscriminat* or unnecessar* or rational* or irrational* or optimal* or optimum or suboptim* or sub-optim*) adj (drug or drugs)).tw,kw. (21044)

78 ((excess* or multipl* or "five or more" or "5 or more") adj2 (medicat* or medicine? or prescrib* or coprescrib* or co-prescrib* or prescription? or coprescription? or co-prescription?)).tw,kw. (12126)

79 ((excess* or multipl* or "five or more" or "5 or more") adj (drug or drugs)).tw,kw. (22344)

80 ((concomitant* or concurrent*) adj2 (medicat* or medicine? or prescrib* or prescription?)).tw,kw. (18814)

81 ((concomitant* or concurrent*) adj (drug or drugs)).tw,kw. (4173)

82 ((omit* or omission?) adj2 (medicat* or medicine? or prescrib* or prescription? or drug or drugs)).tw,kw. (1719)

83 ((under$2 or over$2) adj2 (medicat* or prescrib* or prescription?)).tw,kw. (18032)

84 (underprescri* or overprescri*).tw,kw. (2842)

85 (quality adj2 (prescrib* or prescription?)).tw,kw. (2786)

86 ((multidrug? or multi-drug?) adj2 (pharmacotherap* or pharmaco-therap* or prescrib* or prescription? or regim* or therap* or treatment?)).tw,kw. (12481)

87 (multiple adj (pharmacotherap* or pharmaco-therap*)).tw,kw. (77)

88 ((medication? or medicine? or prescrib* or prescription?) adj3 cascad*).tw,kw. (230)

89 ((medication? or medicine? or prescrib* or prescription?) adj3 continuum*).tw,kw. (142)

90 ((medication? or medicine? or prescrib* or prescription?) adj3 legac*).tw,kw. (114)

91 ((medication? or medicine? or prescrib* or prescription?) adj3 (multi* chronic* or multi* comorbid* or multi* co-morbid* or multimorbid* or multi-morbid*)).tw,kw. (547)

92 (prescrib* adj3 (harm or harmed or harms or harming or harmful*)).tw,kw. (317)

93 STOPP.tw,kw. (1526)

94 "STOPP/START".tw,kw. (670)

95 ((Beers or McLeod or NPS) adj2 criteri*).tw,kw. (2114)

96 "Fit fOR The Aged".tw,kw. (246)

97 ((FORTA or RASP or Pricus) adj2 (criteri* or instrument? or list*)).tw,kw. (76)

98 Assessing Care of Vulnerable Elderly.tw,kw. (3)

99 ACOVE.tw,kw. (193)

100 Medication Appropriateness Index.tw,kw. (399)

101 Medication Regimen Complexity.tw,kw. (423)

102 Prescribing Optimi#ation Method.tw,kw. (6)

103 Systematic Tool to Reduce Inappropriate Prescribing.tw,kw. (29)

104 (strip adj5 (medication? or medicine? or prescrib* or prescription?)).tw,kw. (94)

105 or/67-104 [POLYPHARMACY] (196341)

106 adult/ (12827729)

107 exp aged/ (6489111)

108 aged.ti,kw. (281254)

109 ((age? or year?) adj2 ("65" or "66" or "67" or "68" or "69" or "70" or "71" or "72" or "73" or "74" or "75" or "76" or "77" or "78" or "79" or "80" or "81" or "82" or "83" or "84" or "85" or "86" or "87" or "88" or "89" or "90" or "91" or "92" or "93" or "94" or "95" or "96" or "97" or "98" or "99" or "100")).tw,kw. (1982172)

110 (elderly or geriatric* or gerontolog* or old-age? or senior?).tw,kw. (925937)

111 (older adj2 (adult* or female? or male? or man or men or patient? or person? or people? or population? or wom#n)).tw,kw. (534176)

112 (retiree? or retired or retirement).tw,kw. (46664)

113 (boomer? or babyboomer*).tw,kw. (3450)

114 elderly care/ (40458)

115 exp geriatric care/ (27565)

116 home for the aged/ (12871)

117 (old age adj (facilit* or home? or residen*)).tw,kw. (822)

118 or/106-117 [SENIOR FILTER] (16458300)

119 105 and 118 [POLYPHARMACY - SENIOR FILTER] (89873)

120 exp animal/ or exp animal experimentation/ or exp animal model/ or exp animal experiment/ or nonhuman/ or exp vertebrate/ (53671198)

121 exp human/ or exp human experimentation/ or exp human experiment/ (41652507)

122 120 not 121 (12020450)

123 119 not 122 [ANIMAL-ONLY REMOVED] (89468)

124 editorial.pt. (1195690)

125 letter.pt. not (letter.pt. and randomized controlled trial/) (2212991)

126 123 not (124 or 125) [OPINION PIECES REMOVED] (88139)

127 exp randomized controlled trial/ or controlled clinical trial/ (1401069)

128 "clinical trial (topic)"/ or exp "controlled clinical trial (topic)"/ (287624)

129 (randomi#ed or randomi#ation? or randomly or RCT or placebo*).tw,kw. (3482118)

130 ((singl* or doubl* or trebl* or tripl*) adj (mask* or blind* or dumm*)).tw,kw. (720230)

131 trial.ti. (853918)

132 or/127-131 [RCT FILTER] (4321463)

133 126 and 132 [POLYPHARMACY - SENIORS - RCTs] (17327)

134 133 use emczd [EMBASE RECORDS] (7718)

135 exp polypharmacy/ (22068)

136 (polypharm* or poly-pharm*).ti,ab,kw. (24970)

137 (polymedic* or poly-medic*).ti,ab,kw. (1629)

138 (polyprescri* or poly-prescri*).ti,ab,kw. (23)

139 (polypragmas* or poly-pragmas*).ti,ab,kw. (284)

140 (deprescri* or de-prescri*).ti,ab,kw. (2434)

141 ((cancel* or ceas* or cessation? or discontinu* or halt* or stop* or terminat*) adj2 (medicat* or medicine? or prescrib* or prescription?)).ti,ab,kw. (20128)

142 ((cancel* or ceas* or cessation? or discontinu* or halt* or stop* or terminat*) adj (drug or drugs)).ti,ab,kw. (2673)

143 Inappropriate Prescribing/ (7721)

144 ((appropriate* or inappropriate* or incorrect* or indiscriminat* or unnecessar* or rational* or irrational* or optimal* or optimum or suboptim* or sub-optim*) adj2 (medicat* or medicine? or prescrib* or prescription? or OTC or "over-the-counter" or "behind-the-counter")).ti,ab,kw. (35104)

145 ((appropriate* or inappropriate* or incorrect* or indiscriminat* or unnecessar* or rational* or irrational* or optimal* or optimum or suboptim* or sub-optim*) adj (drug or drugs)).ti,ab,kw. (21042)

146 ((excess* or multipl* or "five or more" or "5 or more") adj2 (medicat* or medicine? or prescrib* or coprescrib* or co-prescrib* or prescription? or coprescription? or co-prescription? or OTC or "over-the-counter")).ti,ab,kw. (12175)

147 ((excess* or multipl* or "five or more" or "5 or more") adj (drug or drugs)).ti,ab,kw. (22344)

148 ((concomitant* or concurrent*) adj2 (medicat* or medicine? or prescrib* or prescription? or OTC or "over-the-counter" or "behind-the-counter")).ti,ab,kw. (18841)

149 ((concomitant* or concurrent*) adj (drug or drugs)).ti,ab,kw. (4173)

150 ((omit* or omission?) adj2 (medicat* or medicine? or prescrib* or prescription? or drug or drugs)).ti,ab,kw. (1719)

151 ((under$2 or over$2) adj2 (medicat* or prescrib* or prescription?)).ti,ab,kw. (18032)

152 (underprescri* or overprescri*).ti,ab,kw. (2842)

153 (quality adj2 (prescrib* or prescription?)).ti,ab,kw. (2786)

154 ((multidrug? or multi-drug?) adj2 (pharmacotherap* or pharmaco-therap* or prescrib* or prescription? or regim* or therap* or treatment?)).ti,ab,kw. (12481)

155 (multiple adj (pharmacotherap* or pharmaco-therap*)).ti,ab,kw. (77)

156 ((medication? or medicine? or prescrib* or prescription?) adj3 cascad*).ti,ab,kw. (230)

157 ((medication? or medicine? or prescrib* or prescription?) adj3 continuum*).ti,ab,kw. (142)

158 ((medication? or medicine? or prescrib* or prescription?) adj3 legac*).ti,ab,kw. (114)

159 ((medication? or medicine? or prescrib* or prescription?) adj3 (multi* chronic* or multi* comorbid* or multi* co-morbid* or multimorbid* or multi-morbid*)).ti,ab,kw. (547)

160 (prescrib* adj3 (harm or harmed or harms or harming or harmful*)).ti,ab,kw. (317)

161 STOPP.ti,ab,kw. (1526)

162 "STOPP/START".ti,ab,kw. (670)

163 ((Beers or McLeod or NPS) adj2 criteri*).ti,ab,kw. (2114)

164 "Fit fOR The Aged".ti,ab,kw. (246)

165 ((FORTA or RASP or Pricus) adj2 (criteri* or instrument? or list*)).ti,ab,kw. (76)

166 Assessing Care of Vulnerable Elderly.ti,ab,kw. (3)

167 ACOVE.ti,ab,kw. (193)

168 Medication Appropriateness Index.ti,ab,kw. (399)

169 Medication Regimen Complexity.ti,ab,kw. (423)

170 Prescribing Optimi#ation Method.ti,ab,kw. (6)

171 Systematic Tool to Reduce Inappropriate Prescribing.ti,ab,kw. (29)

172 (strip adj5 (medication? or medicine? or prescrib* or prescription?)).ti,ab,kw. (94)

173 or/135-172 [POLYPHARMACY] (196476)

174 Adult/ (12827729)

175 exp Aged/ (6489111)

176 aged.ti. (143941)

177 ((age? or year?) adj2 ("65" or "66" or "67" or "68" or "69" or "70" or "71" or "72" or "73" or "74" or "75" or "76" or "77" or "78" or "79" or "80" or "81" or "82" or "83" or "84" or "85" or "86" or "87" or "88" or "89" or "90" or "91" or "92" or "93" or "94" or "95" or "96" or "97" or "98" or "99" or "100")).ti,ab,kw. (1982172)

178 (elderly or geriatric* or gerontolog* or old-age? or senior?).ti,ab,kw. (925916)

179 (older adj2 (adult* or female? or male? or man or men or patient? or person? or people? or population? or wom#n)).ti,ab,kw. (534176)

180 (retiree? or retired or retirement).ti,ab,kw. (46664)

181 (boomer? or babyboomer*).ti,ab,kw. (3449)

182 Health Services for the Aged/ (54126)

183 Homes for the Aged/ (26688)

184 (old age adj (facilit* or home? or residen*)).ti,ab,kw. (822)

185 or/174-184 [SENIOR FILTER] (16396848)

186 173 and 185 [POLYPHARMACY - SENIOR FILTER] (89271)

187 186 use cctr [CENTRAL RECORDS] (5970)

188 66 or 134 or 187 [ALL DATABASES] (18161)

189 limit 188 to yr="2017-current" (4924)

190 remove duplicates from 189 (3551)

191 limit 188 to yr="2011-2016" (5706)

192 remove duplicates from 191 (3692)

193 limit 188 to yr="2001-2010" (4638)

194 remove duplicates from 193 (2806)

195 188 not (189 or 191 or 193) (2893)

196 remove duplicates from 195 (1734)

197 190 or 192 or 194 or 196 [TOTAL UNIQUE RECORDS] (11783)

198 conference abstract.pt. (3840833)

199 journal conference abstract.pt. (157141)

200 197 not (198 or 199) [CONFERENCE ABSTRACTS REMOVED] (9229)

201 197 and (198 or 199) (2554)

202 limit 201 to yr="2018-current" (800)

203 200 or 202 [MOST RECENT 2 YEARS CONFERENCE ABSTRACTS RETAINED] (10029)

***************************

### Key question 2

Database: Embase Classic+Embase <1947 to 2020 July 22>, Ovid MEDLINE(R) ALL <1946 to July 22, 2020>, APA PsycInfo <1806 to July Week 3 2020>, EBM Reviews - Cochrane Database of Systematic Reviews <2005 to July 16, 2020>, EBM Reviews - Database of Abstracts of Reviews of Effects <1st Quarter 2016>, EBM Reviews - NHS Economic Evaluation Database <1st Quarter 2016>

Search Strategy:

--------------------------------------------------------------------------------

1 exp polypharmacy/ (23053)

2 (polypharm* or poly-pharm*).tw,kf. (25308)

3 (polymedic* or poly-medic*).tw,kf. (1604)

4 (polyprescri* or poly-prescri*).tw,kf. (27)

5 (polypragmas* or poly-pragmas*).tw,kf. (276)

6 (deprescri* or de-prescri*).tw,kf. (2346)

7 ((cancel* or ceas* or cessation? or discontinu* or halt* or stop* or terminat*) adj2 (medicat* or medicine? or prescrib* or prescription?)).tw,kf. (19144)

8 ((cancel* or ceas* or cessation? or discontinu* or halt* or stop* or terminat*) adj (drug or drugs)).tw,kf. (1971)

9 Inappropriate Prescribing/ (7589)

10 ((appropriate* or inappropriate* or incorrect* or indiscriminat* or unnecessar* or rational* or irrational* or optimal* or optimum or suboptim* or sub-optim*) adj2 (medicat* or medicine? or prescrib* or prescription? or OTC or "over-the-counter" or "behind-the-counter")).tw,kf. (34768)

11 ((appropriate* or inappropriate* or incorrect* or indiscriminat* or unnecessar* or rational* or irrational* or optimal* or optimum or suboptim* or sub-optim*) adj (drug or drugs)).tw,kf. (20063)

12 ((excess* or multipl* or "five or more" or "5 or more") adj2 (medicat* or medicine? or prescrib* or coprescrib* or co-prescrib* or prescription? or coprescription? or co-prescription? or OTC or "over-the-counter")).tw,kf. (12545)

13 ((excess* or multipl* or "five or more" or "5 or more") adj (drug or drugs)).tw,kf. (21433)

14 ((concomitant* or concurrent*) adj2 (medicat* or medicine? or prescrib* or prescription? or OTC or "over-the-counter" or "behind-the-counter")).tw,kf. (17850)

15 ((concomitant* or concurrent*) adj (drug or drugs)).tw,kf. (4188)

16 ((omit* or omission?) adj2 (medicat* or medicine? or prescrib* or prescription? or drug or drugs)).tw,kf. (1664)

17 ((under$2 or over$2) adj2 (medicat* or prescrib* or prescription?)).tw,kf. (18418)

18 (underprescri* or overprescri*).tw,kf. (2912)

19 (quality adj2 (prescrib* or prescription?)).tw,kf. (2541)

20 ((multidrug? or multi-drug?) adj2 (pharmacotherap* or pharmaco-therap* or prescrib* or prescription? or regim* or therap* or treatment?)).tw,kf. (11914)

21 (multiple adj (pharmacotherap* or pharmaco-therap*)).tw,kf. (85)

22 ((medication? or medicine? or prescrib* or prescription?) adj3 cascad*).tw,kf. (233)

23 ((medication? or medicine? or prescrib* or prescription?) adj3 continuum*).tw,kf. (157)

24 ((medication? or medicine? or prescrib* or prescription?) adj3 legac*).tw,kf. (125)

25 ((medication? or medicine? or prescrib* or prescription?) adj3 (multi* chronic* or multi* comorbid* or multi* co-morbid* or multimorbid* or multi-morbid*)).tw,kf. (511)

26 (prescrib* adj3 (harm or harmed or harms or harming or harmful*)).tw,kf. (322)

27 STOPP.tw,kf. (1459)

28 "STOPP/START".tw,kf. (623)

29 ((Beers or McLeod or NPS) adj2 criteri*).tw,kf. (2149)

30 "Fit fOR The Aged".tw,kf. (245)

31 ((FORTA or RASP or Pricus) adj2 (criteri* or instrument? or list*)).tw,kf. (64)

32 Assessing Care of Vulnerable Elderly.tw,kf. (3)

33 ACOVE.tw,kf. (215)

34 Medication Appropriateness Index.tw,kf. (361)

35 Medication Regimen Complexity.tw,kf. (430)

36 Prescribing Optimi#ation Method.tw,kf. (6)

37 Systematic Tool to Reduce Inappropriate Prescribing.tw,kf. (21)

38 (strip adj5 (medication? or medicine? or prescrib* or prescription?)).tw,kf. (79)

39 or/1-38 [POLYPHARMACY] (192797)

40 Adult/ (12502580)

41 exp Aged/ (6289245)

42 aged.ti,kf. (159469)

43 ((age? or year?) adj2 ("65" or "66" or "67" or "68" or "69" or "70" or "71" or "72" or "73" or "74" or "75" or "76" or "77" or "78" or "79" or "80" or "81" or "82" or "83" or "84" or "85" or "86" or "87" or "88" or "89" or "90" or "91" or "92" or "93" or "94" or "95" or "96" or "97" or "98" or "99" or "100")).tw,kf. (1978844)

44 (elderly or geriatric* or gerontolog* or old-age? or senior?).tw,kf. (974355)

45 (older adj2 (adult* or female? or male? or man or men or patient? or person? or people? or population? or wom#n)).tw,kf. (589504)

46 (retiree? or retired or retirement).tw,kf. (59559)

47 (boomer? or babyboomer*).tw,kf. (5187)

48 Health Services for the Aged/ (53746)

49 Homes for the Aged/ (26096)

50 (old age adj (facilit* or home? or residen*)).tw,kf. (1075)

51 or/40-50 [SENIOR FILTER] (16137803)

52 39 and 51 [POLYPHARMACY - SENIOR FILTER] (86217)

53 exp Animals/ not Humans/ (17865228)

54 52 not 53 [ANIMAL-ONLY REMOVED] (64776)

55 (comment or editorial or news or newspaper article).pt. (2091382)

56 (letter not (letter and randomized controlled trial)).pt. (2211121)

57 54 not (55 or 56) [OPINION PIECES REMOVED] (63667)

58 Choice Behavior/ (258189)

59 (choice? adj2 behavio?r*).tw,kf. (12937)

60 Cooperative Behavior/ (80666)

61 Decision Making/ (403092)

62 decision aid*.tw,kf. (9002)

63 ((guide? or guiding or make or making or makes or made or shar* or support*) adj2 (choice? or choos* or consent* or decid* or decision*)).tw,kf. (595816)

64 Patient Education as Topic/ (179953)

65 Patient Acceptance of Health Care/ (105151)

66 Patient Participation/ (55461)

67 Patient Preference/ (27068)

68 Patient Satisfaction/ (225623)

69 ((engag* or involv* or participat*) adj3 (patient? or person$2 or personally or man or men or "man's" or "men's" or wom#n or "woman's" or "women's")).tw,kf. (384205)

70 exp Patients/px [psychology] (17178)

71 Uncertainty/ (50856)

72 ((accept* or consider* or choice? or choos* or chose? or decid* or decis* or expect* or input* or knowledge* or opinion* or participat* or perspective? or prefer* or respons* or satisf* or uncertain* or understand* or willing*) adj2 (female? or male? or man or men or "man's" or "men's" or patient? or person$2 or personally or wom#n or "woman's" or "women's")).tw,kf. (994096)

73 ((analys#s or valuation? or value? or valuing) adj2 (conjoint or contingent)).tw,kf. (4850)

74 (choice? adj1 (discrete or experiment*)).tw,kf. (9886)

75 ((patient? or person$2 or personally or man or men or "man's" or "men's" or wom#n or "woman's" or "women's") adj (centered or centred or focus*)).tw,kf. (89907)

76 ((patient* or person#2) adj priorit*).tw,kf. (2131)

77 Informed Consent/ (148722)

78 (informed adj (choice* or choos* or consent* or decid* or decision*)).tw,kf. (152939)

79 ((patient? or person$2 or personally or man or men or "man's" or "men's" or wom#n or "woman's" or "women's") adj2 consent*).tw,kf. (37267)

80 ((adher* or nonadher* or non-adher* or inten* or refus* or reject* or uptake or willing*) adj2 (initiat* or intervention? or therap* or treat*)).tw,kf. (304540)

81 (preference? adj1 (elicit* or reveal* or scor* or stated)).tw,kf. (6266)

82 (trade off? or tradeoff? or trade-off?).tw,kf. (71220)

83 Information Seeking Behavior/ (5777)

84 (inform* adj1 seek*).tw,kf. (14859)

85 or/58-84 [PATIENT ACCEPTABILITY/DECISION-MAKING/PARTICIPATION] (3082761)

86 57 and 85 [POLYPHARMACY - SENIORS - PATIENT ACCEPTABILITY/DECISION-MAKING/PARTICIPATION] (10941)

87 limit 86 to english [Limit not valid in DARE,CLEED; records were retained] (10411)

88 limit 86 to french [Limit not valid in CDSR,DARE,CLEED; records were retained] (1004)

89 87 or 88 [LANGUAGE LIMITS APPLIED] (10496)

90 89 use medall [MEDLINE RECORDS] (4258)

91 *polypharmacy/ (7257)

92 (polypharm* or poly-pharm*).ti,kw. (9443)

93 (polymedic* or poly-medic*).ti,kw. (295)

94 (polyprescri* or poly-prescri*).ti,kw. (2)

95 (polypragmas* or poly-pragmas*).ti,kw. (62)

96 (deprescri* or de-prescri*).ti,kw. (1625)

97 ((cancel* or ceas* or cessation? or discontinu* or halt* or stop* or terminat*) adj2 (medicat* or medicine? or prescrib* or prescription?)).ti,kw. (1184)

98 ((cancel* or ceas* or cessation? or discontinu* or halt* or stop* or terminat*) adj (drug or drugs)).ti,kw. (280)

99 exp *inappropriate prescribing/ (4587)

100 ((appropriate* or inappropriate* or incorrect* or indiscriminat* or unnecessar* or rational* or irrational* or optimal* or optimum or suboptim* or sub-optim*) adj2 (medicat* or medicine? or prescrib* or prescription?)).ti,kw. (6588)

101 ((appropriate* or inappropriate* or incorrect* or indiscriminat* or unnecessar* or rational* or irrational* or optimal* or optimum or suboptim* or sub-optim*) adj (drug or drugs)).ti,kw. (2635)

102 ((excess* or multipl* or "five or more" or "5 or more") adj2 (medicat* or medicine? or prescrib* or coprescrib* or co-prescrib* or prescription? or coprescription? or co-prescription?)).ti,kw. (1302)

103 ((excess* or multipl* or "five or more" or "5 or more") adj (drug or drugs)).ti,kw. (4056)

104 ((concomitant* or concurrent*) adj2 (medicat* or medicine? or prescrib* or prescription?)).ti,kw. (814)

105 ((concomitant* or concurrent*) adj (drug or drugs)).ti,kw. (292)

106 ((omit* or omission?) adj2 (medicat* or medicine? or prescrib* or prescription? or drug or drugs)).ti,kw. (147)

107 ((under$2 or over$2) adj2 (medicat* or prescrib* or prescription?)).ti,kw. (1172)

108 (underprescri* or overprescri*).ti,kw. (490)

109 (quality adj2 (prescrib* or prescription?)).ti,kw. (637)

110 ((multidrug? or multi-drug?) adj2 (pharmacotherap* or pharmaco-therap* or prescrib* or prescription? or regim* or therap* or treatment?)).ti,kw. (2871)

111 (multiple adj (pharmacotherap* or pharmaco-therap*)).ti,kw. (2)

112 ((medication? or medicine? or prescrib* or prescription?) adj3 cascad*).ti,kw. (99)

113 ((medication? or medicine? or prescrib* or prescription?) adj3 continuum*).ti,kw. (41)

114 ((medication? or medicine? or prescrib* or prescription?) adj3 legac*).ti,kw. (90)

115 ((medication? or medicine? or prescrib* or prescription?) adj3 (multi* chronic* or multi* comorbid* or multi* co-morbid* or multimorbid* or multi-morbid*)).ti,kw. (107)

116 (prescrib* adj3 (harm or harmed or harms or harming or harmful*)).ti,kw. (31)

117 STOPP.ti,kw. (547)

118 "STOPP/START".ti,kw. (261)

119 ((Beers or McLeod or NPS) adj2 criteri*).ti,kw. (599)

120 "Fit fOR The Aged".ti,kw. (59)

121 ((FORTA or RASP or Pricus) adj2 (criteri* or instrument? or list*)).ti,kw. (20)

122 Assessing Care of Vulnerable Elderly.ti,kw. (0)

123 ACOVE.ti,kw. (46)

124 Medication Appropriateness Index.ti,kw. (68)

125 Medication Regimen Complexity.ti,kw. (225)

126 Prescribing Optimi#ation Method.ti,kw. (4)

127 Systematic Tool to Reduce Inappropriate Prescribing.ti,kw. (9)

128 (strip adj5 (medication? or medicine? or prescrib* or prescription?)).ti,kw. (22)

129 or/91-128 [POLYPHARMACY] (36729)

130 adult/ (12502580)

131 exp aged/ (6289245)

132 aged.ti,kw. (172429)

133 ((age? or year?) adj2 ("65" or "66" or "67" or "68" or "69" or "70" or "71" or "72" or "73" or "74" or "75" or "76" or "77" or "78" or "79" or "80" or "81" or "82" or "83" or "84" or "85" or "86" or "87" or "88" or "89" or "90" or "91" or "92" or "93" or "94" or "95" or "96" or "97" or "98" or "99" or "100")).ti,kw. (53546)

134 (elderly or geriatric* or gerontolog* or old-age? or senior?).ti,kw. (454203)

135 (older adj2 (adult* or female? or male? or man or men or patient? or person? or people? or population? or wom#n)).ti,kw. (204999)

136 (retiree? or retired or retirement).ti,kw. (18163)

137 (boomer? or babyboomer*).ti,kw. (1549)

138 *elderly care/ (20899)

139 exp *geriatric care/ (16758)

140 *home for the aged/ (6414)

141 (old age adj (facilit* or home? or residen*)).ti,kw. (453)

142 or/130-141 [SENIOR FILTER] (15208565)

143 129 and 142 [POLYPHARMACY - SENIOR FILTER] (16997)

144 exp animal/ or exp animal experimentation/ or exp animal model/ or exp animal experiment/ or nonhuman/ or exp vertebrate/ (53434908)

145 exp human/ or exp human experimentation/ or exp human experiment/ (41083279)

146 144 not 145 (12353388)

147 143 not 146 [ANIMAL-ONLY REMOVED] (16975)

148 editorial.pt. (1195233)

149 letter.pt. not (letter.pt. and randomized controlled trial/) (2205797)

150 147 not (148 or 149) [OPINION PIECES REMOVED] (16173)

151 exp *cooperation/ (28495)

152 (choice? adj2 behavio?r*).ti,kw. (2098)

153 *decision making/ (163511)

154 *patient decision making/ (2198)

155 *shared decision making/ (2318)

156 decision aid*.ti,kw. (4465)

157 ((guide? or guiding or make or making or makes or made or shar* or support*) adj2 (choice? or choos* or consent* or decid* or decision*)).ti,kw. (122582)

158 *patient education/ (71856)

159 *patient attitude/ (34754)

160 *patient participation/ (26208)

161 *patient preference/ (10019)

162 *patient satisfaction/ (58367)

163 ((engag* or involv* or participat*) adj3 (patient? or person$2 or personally or man or men or "man's" or "men's" or wom#n or "woman's" or "women's")).ti,kw. (25214)

164 *uncertainty/ (15620)

165 ((accept* or consider* or choice? or choos* or chose? or decid* or decis* or expect* or input* or knowledge* or opinion* or participat* or perspective? or prefer* or respons* or satisf* or uncertain* or understand* or willing*) adj2 (female? or male? or man or men or "man's" or "men's" or patient? or person$2 or personally or wom#n or "woman's" or "women's")).ti,kw. (126574)

166 ((analys#s or valuation? or value? or valuing) adj2 (conjoint or contingent)).ti,kw. (2152)

167 (choice? adj1 (discrete or experiment*)).ti,kw. (3901)

168 ((patient? or person$2 or personally or man or men or "man's" or "men's" or wom#n or "woman's" or "women's") adj (centered or centred or focus*)).ti,kw. (25835)

169 ((patient* or person#2) adj priorit*).ti,kw. (474)

170 *informed consent/ (38895)

171 (informed adj (choice* or choos* or consent* or decid* or decision*)).ti,kw. (22066)

172 ((patient? or person$2 or personally or man or men or "man's" or "men's" or wom#n or "woman's" or "women's") adj2 consent*).ti,kw. (1545)

173 ((adher* or nonadher* or non-adher* or inten* or refus* or reject* or uptake or willing*) adj2 (initiat* or intervention? or therap* or treat*)).ti,kw. (36446)

174 (preference? adj1 (elicit* or reveal* or scor* or stated)).ti,kw. (1092)

175 (trade off? or tradeoff? or trade-off?).ti,kw. (13313)

176 *information seeking/ (4763)

177 (inform* adj1 seek*).ti,kw. (3833)

178 or/151-177 [PATIENT ACCEPTABILITY/DECISION-MAKING/PARTICIPATION] (684252)

179 150 and 178 [POLYPHARMACY - SENIORS - PATIENT ACCEPTABILITY/DECISION-MAKING/PARTICIPATION] (546)

180 limit 179 to english [Limit not valid in DARE,CLEED; records were retained] (505)

181 limit 179 to french [Limit not valid in CDSR,DARE,CLEED; records were retained] (10)

182 180 or 181 [LANGUAGE LIMITS APPLIED] (510)

183 conference abstract.pt. (3824036)

184 182 not 183 (478)

185 182 and 183 (32)

186 limit 185 to yr="2018-current" [Limit not valid in DARE; records were retained] (16)

187 184 or 186 [MOST RECENT 2 YRS CONFERENCE ABSTRACTS RETAINED] (494)

188 187 use emczd [EMBASE RECORDS] (310)

189 polypharmacy/ (22748)

190 (polypharm* or poly-pharm*).tw. (24765)

191 (polymedic* or poly-medic*).tw. (1592)

192 (polyprescri* or poly-prescri*).tw. (27)

193 (polypragmas* or poly-pragmas*).tw. (276)

194 (deprescri* or de-prescri*).tw. (2230)

195 ((cancel* or ceas* or cessation? or discontinu* or halt* or stop* or terminat*) adj2 (medicat* or medicine? or prescrib* or prescription?)).tw. (19120)

196 ((cancel* or ceas* or cessation? or discontinu* or halt* or stop* or terminat*) adj (drug or drugs)).tw. (1966)

197 exp inappropriate prescribing/ (8869)

198 ((appropriate* or inappropriate* or incorrect* or indiscriminat* or unnecessar* or rational* or irrational* or optimal* or optimum or suboptim* or sub-optim*) adj2 (medicat* or medicine? or prescrib* or prescription? or OTC or "over-the-counter" or "behind-the-counter")).tw. (34537)

199 ((appropriate* or inappropriate* or incorrect* or indiscriminat* or unnecessar* or rational* or irrational* or optimal* or optimum or suboptim* or sub-optim*) adj (drug or drugs)).tw. (19928)

200 ((excess* or multipl* or "five or more" or "5 or more") adj2 (medicat* or medicine? or prescrib* or coprescrib* or co-prescrib* or prescription? or coprescription? or co-prescription? or OTC or "over-the-counter")).tw. (12535)

201 ((excess* or multipl* or "five or more" or "5 or more") adj (drug or drugs)).tw. (21335)

202 ((concomitant* or concurrent*) adj2 (medicat* or medicine? or prescrib* or prescription? or OTC or "over-the-counter" or "behind-the-counter")).tw. (17840)

203 ((concomitant* or concurrent*) adj (drug or drugs)).tw. (4175)

204 ((omit* or omission?) adj2 (medicat* or medicine? or prescrib* or prescription? or drug or drugs)).tw. (1661)

205 ((under$2 or over$2) adj2 (medicat* or prescrib* or prescription?)).tw. (18414)

206 (underprescri* or overprescri*).tw. (2896)

207 (quality adj2 (prescrib* or prescription?)).tw. (2537)

208 ((multidrug? or multi-drug?) adj2 (pharmacotherap* or pharmaco-therap* or prescrib* or prescription? or regim* or therap* or treatment?)).tw. (11890)

209 (multiple adj (pharmacotherap* or pharmaco-therap*)).tw. (85)

210 ((medication? or medicine? or prescrib* or prescription?) adj3 cascad*).tw. (231)

211 ((medication? or medicine? or prescrib* or prescription?) adj3 continuum*).tw. (155)

212 ((medication? or medicine? or prescrib* or prescription?) adj3 legac*).tw. (125)

213 ((medication? or medicine? or prescrib* or prescription?) adj3 (multi* chronic* or multi* comorbid* or multi* co-morbid* or multimorbid* or multi-morbid*)).tw. (509)

214 (prescrib* adj3 (harm or harmed or harms or harming or harmful*)).tw. (322)

215 STOPP.tw. (1451)

216 "STOPP/START".tw. (612)

217 ((Beers or McLeod or NPS) adj2 criteri*).tw. (2130)

218 "Fit fOR The Aged".tw. (245)

219 ((FORTA or RASP or Pricus) adj2 (criteri* or instrument? or list*)).tw. (62)

220 Assessing Care of Vulnerable Elderly.tw. (3)

221 ACOVE.tw. (212)

222 Medication Appropriateness Index.tw. (360)

223 Medication Regimen Complexity.tw. (424)

224 Prescribing Optimi#ation Method.tw. (6)

225 Systematic Tool to Reduce Inappropriate Prescribing.tw. (21)

226 (strip adj5 (medication? or medicine? or prescrib* or prescription?)).tw. (79)

227 or/189-226 [POLYPHARMACY] (192050)

228 limit 227 to ("380 aged <age 65 yrs and older>" or "390 very old <age 85 yrs and older>") [Limit not valid in Embase,Ovid MEDLINE(R),Ovid MEDLINE(R) Daily Update,Ovid MEDLINE(R) In-Process,Ovid MEDLINE(R) Publisher,CDSR,DARE,CLEED; records were retained] (183213)

229 aged.ti. (151176)

230 ((age? or year?) adj2 ("65" or "66" or "67" or "68" or "69" or "70" or "71" or "72" or "73" or "74" or "75" or "76" or "77" or "78" or "79" or "80" or "81" or "82" or "83" or "84" or "85" or "86" or "87" or "88" or "89" or "90" or "91" or "92" or "93" or "94" or "95" or "96" or "97" or "98" or "99" or "100")).tw. (1978730)

231 (elderly or geriatric* or gerontolog* or old-age? or senior?).tw. (953867)

232 (older adj2 (adult* or female? or male? or man or men or patient? or person? or people? or population? or wom#n)).tw. (587459)

233 (retiree? or retired or retirement).tw. (59308)

234 (boomer? or babyboomer*).tw. (5164)

235 elder care/ (4559)

236 nursing homes/ (94061)

237 (old age adj (facilit* or home? or residen*)).tw. (952)

238 or/229-237 (3225449)

239 227 and 238 (40565)

240 228 or 239 [POLYPHARMACY - SENIOR FILTER] (183990)

241 exp Choice Behavior/ (482339)

242 (choice? adj2 behavio?r*).tw. (12804)

243 Cooperation/ (58158)

244 Decision Making/ (403092)

245 Group Decision Making/ (3189)

246 decision aid*.tw. (8837)

247 ((guide? or guiding or make or making or makes or made or shar* or support*) adj2 (choice? or choos* or consent* or decid* or decision*)).tw. (589282)

248 Client Education/ (4022)

249 Client Participation/ (2263)

250 exp Client Attitudes/ (22196)

251 ((engag* or involv* or participat*) adj3 (patient? or person$2 or personally or man or men or "man's" or "men's" or wom#n or "woman's" or "women's")).tw. (383238)

252 exp Uncertainty/ (51800)

253 ((accept* or consider* or choice? or choos* or chose? or decid* or decis* or expect* or input* or knowledge* or opinion* or participat* or perspective? or prefer* or respons* or satisf* or uncertain* or understand* or willing*) adj2 (female? or male? or man or men or "man's" or "men's" or patient? or person$2 or personally or wom#n or "woman's" or "women's")).tw. (991305)

254 ((analys#s or valuation? or value? or valuing) adj2 (conjoint or contingent)).tw. (4726)

255 (choice? adj1 (discrete or experiment*)).tw. (9806)

256 ((patient? or person$2 or personally or man or men or "man's" or "men's" or wom#n or "woman's" or "women's") adj (centered or centred or focus*)).tw. (87534)

257 ((patient* or person#2) adj priorit*).tw. (2114)

258 Informed Consent/ (148722)

259 (informed adj (choice* or choos* or consent* or decid* or decision*)).tw. (152005)

260 ((patient? or person$2 or personally or man or men or "man's" or "men's" or wom#n or "woman's" or "women's") adj2 consent*).tw. (37257)

261 ((adher* or nonadher* or non-adher* or inten* or refus* or reject* or uptake or willing*) adj2 (initiat* or intervention? or therap* or treat*)).tw. (303909)

262 (preference? adj1 (elicit* or reveal* or scor* or stated)).tw. (6162)

263 (trade off? or tradeoff? or trade-off?).tw. (70549)

264 Information Seeking/ (7182)

265 (inform* adj1 seek*).tw. (14631)

266 or/241-265 [PATIENT ACCEPTABILITY/DECISION-MAKING/PARTICIPATION] (2830918)

267 240 and 266 [POLYPHARMACY - SENIORS - PATIENT ACCEPTABILITY/DECISION-MAKING/PARTICIPATION] (23563)

268 limit 267 to english [Limit not valid in DARE,CLEED; records were retained] (22388)

269 limit 267 to french [Limit not valid in CDSR,DARE,CLEED; records were retained] (1901)

270 268 or 269 [LANGUAGE LIMITS APPLIED] (22591)

271 270 use medall,emczd,coch,dare,cleed (22081)

272 270 not 271 [PSYCINFO RECORDS] (510)

273 exp polypharmacy/ (23053)

274 (polypharm* or poly-pharm*).ti,ab,kw. (26073)

275 (polymedic* or poly-medic*).ti,ab,kw. (1612)

276 (polyprescri* or poly-prescri*).ti,ab,kw. (27)

277 (polypragmas* or poly-pragmas*).ti,ab,kw. (281)

278 (deprescri* or de-prescri*).ti,ab,kw. (2418)

279 ((cancel* or ceas* or cessation? or discontinu* or halt* or stop* or terminat*) adj2 (medicat* or medicine? or prescrib* or prescription?)).ti,ab,kw. (18821)

280 ((cancel* or ceas* or cessation? or discontinu* or halt* or stop* or terminat*) adj (drug or drugs)).ti,ab,kw. (1929)

281 Inappropriate Prescribing/ (7589)

282 ((appropriate* or inappropriate* or incorrect* or indiscriminat* or unnecessar* or rational* or irrational* or optimal* or optimum or suboptim* or sub-optim*) adj2 (medicat* or medicine? or prescrib* or prescription? or OTC or "over-the-counter" or "behind-the-counter")).ti,ab,kw. (34751)

283 ((appropriate* or inappropriate* or incorrect* or indiscriminat* or unnecessar* or rational* or irrational* or optimal* or optimum or suboptim* or sub-optim*) adj (drug or drugs)).ti,ab,kw. (20277)

284 ((excess* or multipl* or "five or more" or "5 or more") adj2 (medicat* or medicine? or prescrib* or coprescrib* or co-prescrib* or prescription? or coprescription? or co-prescription? or OTC or "over-the-counter")).ti,ab,kw. (12491)

285 ((excess* or multipl* or "five or more" or "5 or more") adj (drug or drugs)).ti,ab,kw. (21558)

286 ((concomitant* or concurrent*) adj2 (medicat* or medicine? or prescrib* or prescription? or OTC or "over-the-counter" or "behind-the-counter")).ti,ab,kw. (17033)

287 ((concomitant* or concurrent*) adj (drug or drugs)).ti,ab,kw. (4085)

288 ((omit* or omission?) adj2 (medicat* or medicine? or prescrib* or prescription? or drug or drugs)).ti,ab,kw. (1646)

289 ((under$2 or over$2) adj2 (medicat* or prescrib* or prescription?)).ti,ab,kw. (18123)

290 (underprescri* or overprescri*).ti,ab,kw. (2915)

291 (quality adj2 (prescrib* or prescription?)).ti,ab,kw. (2534)

292 ((multidrug? or multi-drug?) adj2 (pharmacotherap* or pharmaco-therap* or prescrib* or prescription? or regim* or therap* or treatment?)).ti,ab,kw. (11931)

293 (multiple adj (pharmacotherap* or pharmaco-therap*)).ti,ab,kw. (84)

294 ((medication? or medicine? or prescrib* or prescription?) adj3 cascad*).ti,ab,kw. (234)

295 ((medication? or medicine? or prescrib* or prescription?) adj3 continuum*).ti,ab,kw. (155)

296 ((medication? or medicine? or prescrib* or prescription?) adj3 legac*).ti,ab,kw. (124)

297 ((medication? or medicine? or prescrib* or prescription?) adj3 (multi* chronic* or multi* comorbid* or multi* co-morbid* or multimorbid* or multi-morbid*)).ti,ab,kw. (531)

298 (prescrib* adj3 (harm or harmed or harms or harming or harmful*)).ti,ab,kw. (316)

299 STOPP.ti,ab,kw. (1455)

300 "STOPP/START".ti,ab,kw. (627)

301 ((Beers or McLeod or NPS) adj2 criteri*).ti,ab,kw. (2171)

302 "Fit fOR The Aged".ti,ab,kw. (244)

303 ((FORTA or RASP or Pricus) adj2 (criteri* or instrument? or list*)).ti,ab,kw. (62)

304 Assessing Care of Vulnerable Elderly.ti,ab,kw. (2)

305 ACOVE.ti,ab,kw. (215)

306 Medication Appropriateness Index.ti,ab,kw. (359)

307 Medication Regimen Complexity.ti,ab,kw. (433)

308 Prescribing Optimi#ation Method.ti,ab,kw. (6)

309 Systematic Tool to Reduce Inappropriate Prescribing.ti,ab,kw. (21)

310 (strip adj5 (medication? or medicine? or prescrib* or prescription?)).ti,ab,kw. (78)

311 or/273-310 [POLYPHARMACY] (191946)

312 Adult/ (12502580)

313 exp Aged/ (6289245)

314 aged.ti. (151176)

315 ((age? or year?) adj2 ("65" or "66" or "67" or "68" or "69" or "70" or "71" or "72" or "73" or "74" or "75" or "76" or "77" or "78" or "79" or "80" or "81" or "82" or "83" or "84" or "85" or "86" or "87" or "88" or "89" or "90" or "91" or "92" or "93" or "94" or "95" or "96" or "97" or "98" or "99" or "100")).ti,ab,kw. (1969443)

316 (elderly or geriatric* or gerontolog* or old-age? or senior?).ti,ab,kw. (974880)

317 (older adj2 (adult* or female? or male? or man or men or patient? or person? or people? or population? or wom#n)).ti,ab,kw. (585446)

318 (retiree? or retired or retirement).ti,ab,kw. (58919)

319 (boomer? or babyboomer*).ti,ab,kw. (5148)

320 Health Services for the Aged/ (53746)

321 Homes for the Aged/ (26096)

322 (old age adj (facilit* or home? or residen*)).ti,ab,kw. (950)

323 or/312-322 [SENIOR FILTER] (16124500)

324 311 and 323 [POLYPHARMACY - SENIOR FILTER] (85635)

325 Choice Behavior/ (258189)

326 (choice? adj2 behavio?r*).ti,ab,kw. (10701)

327 Cooperative Behavior/ (80666)

328 Decision Making/ (403092)

329 decision aid*.ti,ab,kw. (9014)

330 ((guide? or guiding or make or making or makes or made or shar* or support*) adj2 (choice? or choos* or consent* or decid* or decision*)).ti,ab,kw. (581120)

331 Patient Education as Topic/ (179953)

332 Patient Acceptance of Health Care/ (105151)

333 Patient Participation/ (55461)

334 Patient Preference/ (27068)

335 Patient Satisfaction/ (225623)

336 ((engag* or involv* or participat*) adj3 (patient? or person$2 or personally or man or men or "man's" or "men's" or wom#n or "woman's" or "women's")).ti,ab,kw. (380259)

337 exp Patients/px [psychology] (17178)

338 Uncertainty/ (50856)

339 ((accept* or consider* or choice? or choos* or chose? or decid* or decis* or expect* or input* or knowledge* or opinion* or participat* or perspective? or prefer* or respons* or satisf* or uncertain* or understand* or willing*) adj2 (female? or male? or man or men or "man's" or "men's" or patient? or person$2 or personally or wom#n or "woman's" or "women's")).ti,ab,kw. (980023)

340 ((analys#s or valuation? or value? or valuing) adj2 (conjoint or contingent)).ti,ab,kw. (4892)

341 (choice? adj1 (discrete or experiment*)).ti,ab,kw. (9873)

342 ((patient? or person$2 or personally or man or men or "man's" or "men's" or wom#n or "woman's" or "women's") adj (centered or centred or focus*)).ti,ab,kw. (88863)

343 ((patient* or person#2) adj priorit*).ti,ab,kw. (2179)

344 Informed Consent/ (148722)

345 (informed adj (choice* or choos* or consent* or decid* or decision*)).ti,ab,kw. (152253)

346 ((patient? or person$2 or personally or man or men or "man's" or "men's" or wom#n or "woman's" or "women's") adj2 consent*).ti,ab,kw. (37079)

347 ((adher* or nonadher* or non-adher* or inten* or refus* or reject* or uptake or willing*) adj2 (initiat* or intervention? or therap* or treat*)).ti,ab,kw. (291240)

348 (preference? adj1 (elicit* or reveal* or scor* or stated)).ti,ab,kw. (6156)

349 (trade off? or tradeoff? or trade-off?).ti,ab,kw. (70971)

350 Information Seeking Behavior/ (5777)

351 (inform* adj1 seek*).ti,ab,kw. (14198)

352 or/325-351 [PATIENT ACCEPTABILITY/DECISION-MAKING/PARTICIPATION] (3054549)

353 324 and 352 [POLYPHARMACY - SENIORS - PATIENT ACCEPTABILITY/DECISION-MAKING/PARTICIPATION] (13761)

354 353 use coch,dare,cleed [COCHRANE RECORDS] (8)

355 90 or 188 or 272 or 354 [ALL DATABASES] (5086)

356 remove duplicates from 355 (4543)

***************************
