# Supplementary material for: Interventions to address potentially inappropriate prescriptions and over-the-counter medication use among adults 65 years and older in primary care settings: protocol for a systematic review
Source: Syst Rev. 2022 Oct 20;11:225. doi: 10.1186/s13643-022-02044-w (PMC9585747; doi:10.1186/s13643-022-02044-w)
Supplement: Supplementary file 3 — Additional file 3. List of grey literature relevant websites. [file 13643_2022_2044_MOESM3_ESM.docx]

## Additional file 3: List of grey literature relevant websites

- Age UK: [www.ageuk.org.uk](http://www.ageuk.org.uk)
- Australian Deprescribing Network (ADeN): [www.australiandeprescribingnetwork.com/au/](https://australiandeprescribingnetwork.com/au/)
- Canadian Agency for Drugs and Technologies in Health (CADTH): [www.cadth.ca](http://www.cadth.ca)
- Canadian Deprescribing Network: [www.deprescribingnetwork.ca](https://www.deprescribingnetwork.ca/)
- Canadian Institute for Health Information (CIHI): [www.cihi.ca](http://www.cihi.ca)
- Choosing Wisely: www.choosingwisely.org
- College of Physicians and Surgeons of Alberta: www.cpsa.ca
- deprescribing.org: [www.deprescribing.org](http://www.deprescribing.org)
- English Deprescribing Network (EDeN): [www.sps.nhs.uk/networks/english-deprescribing-network](http://www.sps.nhs.uk/networks/english-deprescribing-network) & <https://twitter.com/EDeprescribeN>
- Lown Institute: https://lowninstitute.org/projects/medication-overload-how-the-drive-to-prescribe-is-harming-older-americans/
- Network of Northern European Researchers in Deprescribing: <https://twitter.com/NDeprescribing>
- Ontario Ministry of Health and Long-Term Care: [www.health.gov.on.ca](http://www.health.gov.on.ca/en/)
- Ontario Pharmacists Association: [www.opatoday.com](http://www.opatoday.com)
- Ontario Pharmacy Evidence Network: [www.open-pharmacy-research.ca](http://www.open-pharmacy-research.ca)
- The Clinical Decision Support Lab: [www.mchi.mcgill.ca/index.php/the-clinical-decision-support-lab/](http://www.mchi.mcgill.ca/index.php/the-clinical-decision-support-lab/)
- The Institute for Safe Medication Practices Canada: [www.ismp-canada.org](https://www.ismp-canada.org/)
- US Describing Research Network: <https://deprescribingresearch.org/>
