# Supplementary material for: Interventions to address potentially inappropriate prescriptions and over-the-counter medication use among adults 65 years and older in primary care settings: protocol for a systematic review
Source: Syst Rev. 2022 Oct 20;11:225. doi: 10.1186/s13643-022-02044-w (PMC9585747; doi:10.1186/s13643-022-02044-w)
Supplement: Supplementary file 4 — Additional file 4. Definitions. [file 13643_2022_2044_MOESM4_ESM.docx]

## Additional file 4: Definitions

| Patient-level intervention | An intervention where the investigators identified target medications to address potentially inappropriate medication use and implemented the process with a patient or participant (investigator-led intervention). The investigators then made recommendations to the patient or participant’s prescriber(s) accordingly (adapted from [Page et al. 2016](https://www.ncbi.nlm.nih.gov/pmc/articles/PMC5338123/)). |
| --- | --- |
| Prescriber-level or Population-level intervention | An intervention that is delivered to population of providers or prescribers, and the same intervention is delivered to every member of this population. E.g., education interventions to prescribers (adapted from [Page et al. 2016](https://www.ncbi.nlm.nih.gov/pmc/articles/PMC5338123/)). |
| Explicit review or intervention | An intervention that aims to identify specific medications or medication combinations that might be inappropriate, and that is criterion-based. E.g., list of drugs, drugs classes and dosages known to cause harmful effects, Beers List, McLeod, STOPP/START criteria, PRISCUS (adapted from <https://www.pcne.org/upload/files/100_2015_Workshop_1_Intro-2.pdf> ). |
| Implicit review or intervention | An intervention that aims at identifying potentially inappropriate medications in general, and that relies on expert professional judgement while focusing on the patient and addresses the entire medication regimen. E.g., statements like “is there an indication for the drug?”, MAI, Lipton Criteria (adapted from <https://www.pcne.org/upload/files/100_2015_Workshop_1_Intro-2.pdf> ). |
| Medication reconciliation | The process of comparing what a patient should be taking (i.e., what has been prescribed to them by a healthcare provider) to what they are actually taking. This is done to avoid medication errors and may aid in deprescribing some drugs. |
| Deprescribing drugs | The planned process of reducing, or stopping a medication that may no longer be beneficial, or that may be causing harm. Done in consultation with a healthcare provider. |
| Medication review | The process by which a healthcare provider examines the patient or participant’s current (and/or previous) list of over-the-counter and prescription drugs. This can be facilitated with a computerized program. |
| Intervention attribute | Specific components that make up an intervention. For the purpose of this work, intervention attributes include: intervention setting, type of healthcare provider, and tool. |
| First contact care provider | A licensed healthcare provider who is delivering the intervention. For the purpose of this work, the most common types are: pharmacists, nurses, and general practitioners. |
| Setting | Setting in which the patients are seen and the intervention is delivered (e.g., clinic, pharmacy). |
| Computerized software tool | A computer program that scans the patients electronic medical record to identify current (and/or previous) over-the-counter and prescription drugs. |
| Validated tool | The tool that is used to do the assessment was previously validated and results (e.g., sensitivity, specificity) can be found in a peer-reviewed publication. |
| Inappropriate prescriptions use | The use of prescriptions medication, alone or in combination with other prescription medication and/or over-the-counter medication, that is inappropriate (as determined by the provider’s clinical judgment or by a tool) for the patient’s health state and could lead to drug-related adverse events. |
| Inappropriate over-the-counter drug use | The use of over-the-counter medication, alone or in combination with other prescription medication and/or over-the-counter medication, that is inappropriate (as determined by the provider’s clinical judgment or by a tool) for the patient’s health state and could lead to drug-related adverse events. |
| Over-the-counter drugs | Drugs that may be obtained directly from a pharmacist without a prescription from a physician or nurse practitioner. |
| Prescription drugs | Drugs that require a prescription from a healthcare provider. |
