# Supplementary material for: Interventions to address potentially inappropriate prescriptions and over-the-counter medication use among adults 65 years and older in primary care settings: protocol for a systematic review
Source: Syst Rev. 2022 Oct 20;11:225. doi: 10.1186/s13643-022-02044-w (PMC9585747; doi:10.1186/s13643-022-02044-w)
Supplement: Supplementary file 5 — Additional file 5. Stakeholder review and feedback. [file 13643_2022_2044_MOESM5_ESM.docx]

## Additional file 5: Stakeholder review and feedback

**Reviewers**: Dr. Jamie Falk (Centre of Aging), Dr. Herman Chow (Canadian Geriatrics Society), Dr. James Silvius (Canadian Geriatrics Society), Dr. Jean Triscott (Canadian Geriatrics Society)

| **Question 1:** The Task Force is committed to creating guidelines that enhance equity. Are there additional specific populations that should be considered for this topic, and do you have any concerns or suggestions regarding how specific populations of interest within the background and PICO table have been described? | | |
| --- | --- | --- |
| **Replies** | **Reviewers Response** | **Authors Response** |
| Yes: 2  No: 2 | 1. No concerns about the descriptors provided. I wonder if frailty could be used as a further descriptor of patient populations. If this information is available/separated in the studies captured, I wouldn’t want the chance to observe benefits/harms in this important subgroup of older adults to be missed. This may be part of the separate analyses that are planned.  2. I don’t see reference to indigenous/first nations and this is a population of interest where there may be differences of relevance. It is also highly likely that there will be a paucity of information available. I would also suggest that stratification by gender would be important, both because of the sex differences in medication handling by the body and because women may be a relatively more at-risk population for a variety of reasons. It is acknowledged further on that gender will be included in a sensitivity analysis so it may be captured.  3. Adults 65 years and older in primary care setting is appropriate.    4. Population identified is the one of interest. It is clear and appropriate for the protocol. | 1. Thank you, this will be extracted from included studies under patient characteristics and can be planned for the additional analyses.  2. Thank you, Indigenous/First Nations are not specifically mentioned as a population of interest, but they will be included as a population of interest in they are community-dwelling adults aged 65 years or older. Ethnicity and gender will be considered during sensitivity analyses according to equity considerations.  3. Thank you.  4. Thank you. |
| **Question 2:** Do you have any comments or suggestions related to the eligibility criteria (interventions, comparisons, outcomes) for the research questions? (see relevant tables**: Table 1 – Key question 1 eligibility criteria** and **Table 2 – Key question 2 eligibility criteria**) | | |
| **Replies** | **Reviewers Response** | **Authors Response** |
| Yes: 2  No: 2 | 1a. Depending on what is found, there may be large enough samples to look at benefit/harm of interventions for individual drug classes. This may be of heightened importance for example if there is a difference in how hard a class is to stop or how hard it is to stop meds in a population with a certain condition requiring certain drugs, etc. Is there an a priori plan to deal with this?  1b. Will non-serious ADR be dichotomous (e.g. any non-serious ADRs - yes or no?) or individual ADRs and their %?  1c. Only interventions effective for critical outcomes will be considered for KQ2. Is there consideration for the same to apply to effect seen on important outcomes as well? A difference in one or several of those (especially QoL, ER visits, injurious fall) would still in my mind justify an assessment of acceptability.  1d. For the setting, would geriatric assessment programs be eligible (often separate from primary care, but not necessarily a specialist clinic)?  1e. If LTC studies are accepted because of their applicability to primary care settings, might suggest that if this is the case, that outcomes for subgroups be analyzed? Even if its an intervention that is applicable to the primary care setting, the feasibility of delivering it and the potential successes/harms may be considerably different in LTC vs community populations. I suppose the final GRADE rating may reflect this (e.g. indirectness if there is uncertainty about how this would apply to primary care when done in LTC?)  1f. What's the likelihood of low risk of bias studies since most of the RCTs available likely weren't blinded for participants or clinicians (maybe only outcome assessors)? Would there be a plan to maybe take out the participant blinding question from the RoB assessment for this in order to base separate analyses for RoB on the other criteria?  1g. Lastly, regarding separate analyses, sample size may be worth analyzing (e.g. studies with >150 vs <150 participants) especially since using random effects model which can allow small studies to have disproportionate effect on MA results.  2. In Table 3, interventions are entirely focused on healthcare providers however there have been interventions focused on educating patients – I think it is a gap that interventions focused on patients are not included. I also wonder about interventions that attempt to direct patients to non-pharmacologic management and away from medications where appropriate. I don’t see that referenced amongst interventions.  3. Criteria and inclusion, exclusion is appropriate. Study design: RCTs (individual or cluster, non-randomized experimental study, or observational study design.  4. Eligibility is complete, clear, and logical. | 1a. Thank you, we will be extracting this information if provided in the included studies and this will be considered as a potential sensitivity analysis for drug type targeted or intervention type.  1b. We will be relying on how the study authors reported outcome data. We will transform data from the included studies to ensure consistent presentation and synthesis of the results across the studies. We may contact study authors if we request more information or data.  1c. Only outcomes considered critical will be considered. Following GRADE guidance, critical outcomes are the primary factors for influencing a recommendation and are used to determine the overall quality of evidence supporting a recommendation.  1d. If these are not specialist clinics and are relevant to primary care where the intervention is delivered or initiated by first contact care providers, then we would include this setting. The article would have to meet the other inclusion criteria as well.  1e. Thank you, yes this will be considered in the sensitivity analyses of different types of providers/settings and GRADE assessments.  1f. Thank you, RoB assessments do allow for some flexibility, and we will keep this in mind during assessments and the possibility or impossibility of blinding in certain scenarios.  1g. Thank you, this will be considered during the GRADE assessments and the imprecision domain.  2. Thank you, we will be considering educational interventions that include care providers and their patients. However, we are not interested in educational interventions limited to only patients as this review and guideline is focused on primary care providers and potentially inappropriate prescribing.  3. Thank you.  4. Thank you. |
| **Question 3:** Do the research questions address the clinically important issues? | | |
| **Replies** | **Reviewers Response** | **Authors Response** |
| Yes: 4  No: 0 | 1. Yes, definitely the big clinically important issues are addressed. Minor point: See above point 3. In addition, will resource utilization be addressed? This is difficult to analyze, likely won’t be an outcome in studies included, and will vary greatly depending on study setting and intervention type, but this is an important question I think in order for implementation of eventual recommendations to be feasible. Is there consideration of a narrative review of this question?  2. No comments. I think these are the right questions.  3. Yes. Outcomes: quantitative information 1. All-cause mortality, 2. Hospitalization, 3. Adverse drug reactions, 4. QoL, 5. ER visits. May be important to include literature on falls, delirium, confusion, urinary incontinence, and immobility.  4. Agree with the identified questions, they address the issues. | 1. Thank you. We had previously considered resource utilization and how to address this in the review and guideline. This will be considered during the guideline development using the GRADE Evidence to Decision Framework which includes a domain on resources, cost effectiveness, and feasibility.  2. Thank you.  3. Thank you.  4. Thank you. |
| **Question 4:** Are there any important sources of studies (i.e databases or organizational websites) that we did not include that should be considered in our review? If yes, please provide additional sources. | | |
| **Replies** | **Reviewers Response** | **Authors Response** |
| Yes: 0  No: 4 | 1. The proposed grey literature search is widely encompassing  2. You have captured the key ones. Other deprescribing networks are included in the deprescribing.org website and may have additional links.  3. The Care & Elderly Health Guide can be a resource. Triscott JAC, Dobbs B, Charles L, Huang J, Moores D, Tian PGJ. The Care-of-the-Elderly Health Guide. J Prim Care Community Health. 2021 Jan-Dec;12:21501327211044058. doi: 10.1177/21501327211044058. PMID: 34486428; PMCID: PMC8424605.  4. No. | Thank you to all the reviewers and we will consider these sources during the review. |
| **Question 5:** Are there specific reports or publications of research studies, or ongoing studies that might fit the inclusion criteria, that the Task Force should consider? | | |
| **Replies** | **Reviewers Response** | **Authors Response** |
| Yes: 2  No: 2 | 1. MinMed pragmatic trial? – won’t likely have results in time (https://pragmatictrials.ca/minmed/)  2. There will be work coming from CaDeN but I am uncertain as to where it is in process. It relates to SaferMedsNL and to work done in Manitoba. Justin Turner is the contact.  3. Triscott JAC, Dobbs B, Charles L, Huang J, Moores D, Tian PGJ. The Care-of-the-Elderly Health Guide. J Prim Care Community Health. 2021 Jan-Dec;12:21501327211044058. doi: 10.1177/21501327211044058. PMID: 34486428; PMCID: PMC8424605.  4. No. | Thank you to all the reviewers and we will consider these reports and publications during the review. |
| **Question 6:** Do you have any major concerns about the protocol that we should address? | | |
| **Replies** | **Reviewers Response** | **Authors Response** |
| Yes: 0  No: 4 | 1. No comment  2. No comment  3. No comment  4. Thorough. No major concerns. | Thank you to all the reviewers. |
